# Sources and control of impurity during one-pot enzymatic production of dehydroepiandrosterone

**DOI:** 10.1007/s00253-024-13221-3

**Published:** 2024-06-29

**Authors:** Jiawei Dai, Zheyi Wu, Zebin Liu, Chen Li, Linjiang Zhu, Hanchi Chen, Xiaolong Chen

**Affiliations:** 1 Institute of Fermentation Engineering, Zhejiang University of Technology, Hangzhou, 310014 China; 2 College of Biotechnology and Bioengineering, Zhejiang University of Technology, Hangzhou, 310014 China

**Keywords:** DHEA, Impurity control strategy, Promiscuous enzymes, Ketosteroid isomerase activity

## Abstract

**Abstract:**

Dehydroepiandrosterone (DHEA) has a promising market due to its capacity to regulate human hormone levels as well as preventing and treating various diseases. We have established a chemical esterification coupled biocatalytic-based scheme by lipase-catalyzed 4-androstene-3,17-dione (4-AD) hydrolysis to obtain the intermediate product 5-androstene-3,17-dione (5-AD), which was then asymmetrically reduced by a ketoreductase from *Sphingomonas wittichii* (SwiKR). Co-enzyme required for KR is regenerated by a glucose dehydrogenase (GDH) from *Bacillus subtilis*. This scheme is more environmentally friendly and more efficient than the current DHEA synthesis pathway. However, a significant amount of 4-AD as by-product was detected during the catalytic process. Focused on the control of by-products, we investigated the source of 4-AD and identified that it is mainly derived from the isomerization activity of SwiKR and GDH. Increasing the proportion of glucose in the catalytic system as well as optimizing the catalytic conditions drastically reduced 4-AD from 24.7 to 6.5% of total substrate amount, and the final yield of DHEA achieved 40.1 g/L. Furthermore, this is the first time that both SwiKR and GDH have been proved to be promiscuous enzymes with dehydrogenase and ketosteroid isomerase (KSI) activities, expanding knowledge of the substrate diversity of the short-chain dehydrogenase family enzymes.

**Key points:**

*• A strategy of coupling lipase, ketoreductase, and glucose dehydrogenase in producing DHEA from 4-AD*

*• Both SwiKR and GDH are identified with ketosteroid isomerase activity.*

*• Development of catalytic strategy to control by-product and achieve highly selective DHEA production*

**Graphical abstract:**

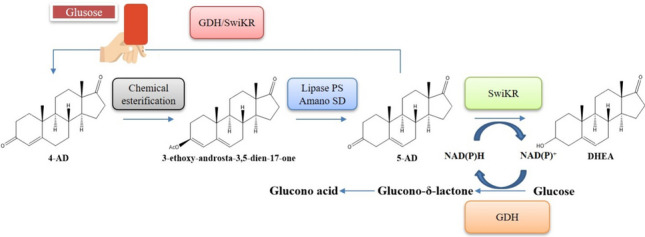

**Supplementary Information:**

The online version contains supplementary material available at 10.1007/s00253-024-13221-3.

## Introduction

Dehydroepiandrosterone (DHEA), an important endogenous steroid hormone, is a precursor substance for many hormones required by the body, such as testosterone, estrogen, progesterone, aldosterone, and cortisol. The production of DHEA by the human adrenal glands begins at puberty, peaks at the age of 20, and begins to decline after age 25 (Flynn et al. 1999; Genazzani et al. 2003; Wierman and Kiseljak-Vassiliades 2022). Its concentration in human blood has been found to be inversely correlated with the incidence of many age-related diseases and cancers (Artini et al. 2012; group 2002; Ihenacho et al. 2022; Jia et al. 2020; Zhang et al. 2023); therefore, it has been promoted as mother hormone, miracle pill, fountain of youth, and antidote to age (Sahu et al. 2020). DHEA has been extensively studied for its medical importance, including the treatment of COVID-19 (Tomo et al. 2022; Tomo et al. 2021), Alzheimer’s disease (Pan et al. 2019), cardiovascular disease (Nippoldt and Nair 1998), menopausal disorders (Gajarawala et al. 2019; Huang et al. 2019; Huang and Wu 2018), as well as breast and prostate cancer (Chatterton 2022; Ke et al. 2013).

**Scheme 1:**
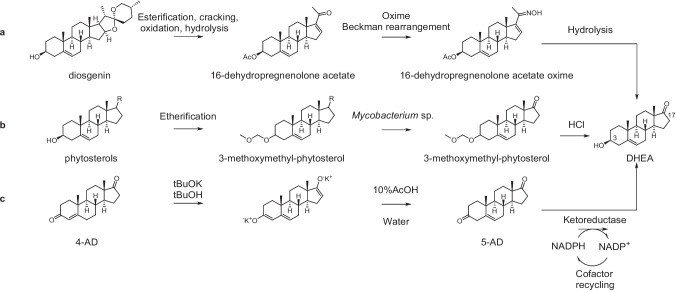
Chemical and biosynthesis preparation of DHEA

Scheme 1a illustrates the primary industrial production pathway of DHEA using diosgenin as the raw material, obtained through high-temperature esterification, cracking, oxidation, alkaline hydrolysis, and oxime followed by Beckman rearrangement (Hosoda et al. 1973). However, this chemical synthesis pathway involves at least three steps, along with a series of protection and deprotection steps, to achieve stereoselective and regioselective reduction at the C3 position. Additionally, it necessitates the extensive use of organic reagents such as benzene, halogenated alkanes, or pyridine, which does not align with current environmental standards. The biosynthesis of DHEA shown as Scheme 1b has also been reported in a process involving the degradation of the C17 side chain of phytosterols by *Mycobacterium* sp., and the substrate can be obtained in large quantities from by-products or waste products of the vegetable oil refining industry (Zhao et al. 2021). The biosynthesis of DHEA is hindered by its low synthetic scale and conversion rate, with only 16.33 g of DHEA produced per liter and a DHEA yield of 92.65%. Consequently, it is not feasible for large-scale production at present (Zhou et al. 2018).

In contrast, biocatalytic asymmetric ketone reduction reactions with high enantioselectivity, mild reaction conditions, and environmental friendliness have become an ideal approach for the synthesis DHEA (Prier and Kosjek 2019). Currently, the process involves a chemical isomerization step using tert-butanol and tert-butanol potassium to obtain the intermediate product 5-AD, which is then asymmetrically reduced by a dual enzyme coupling of KR and coenzyme cycle enzyme belonging to the short-chain dehydrogenase family (SDRs) to obtain the production DHEA (Scheme 1c) (Fryszkowska et al. 2016). However, this isomerization reaction requires the consumption of large amounts of tert-butanol and potassium tert-butoxide to produce the intermediate product 5-AD, with a final reaction yield of about 80% and 90% purity, and is prone to produce 4-AD which is difficult to remove in subsequent production.

**Scheme 2:**
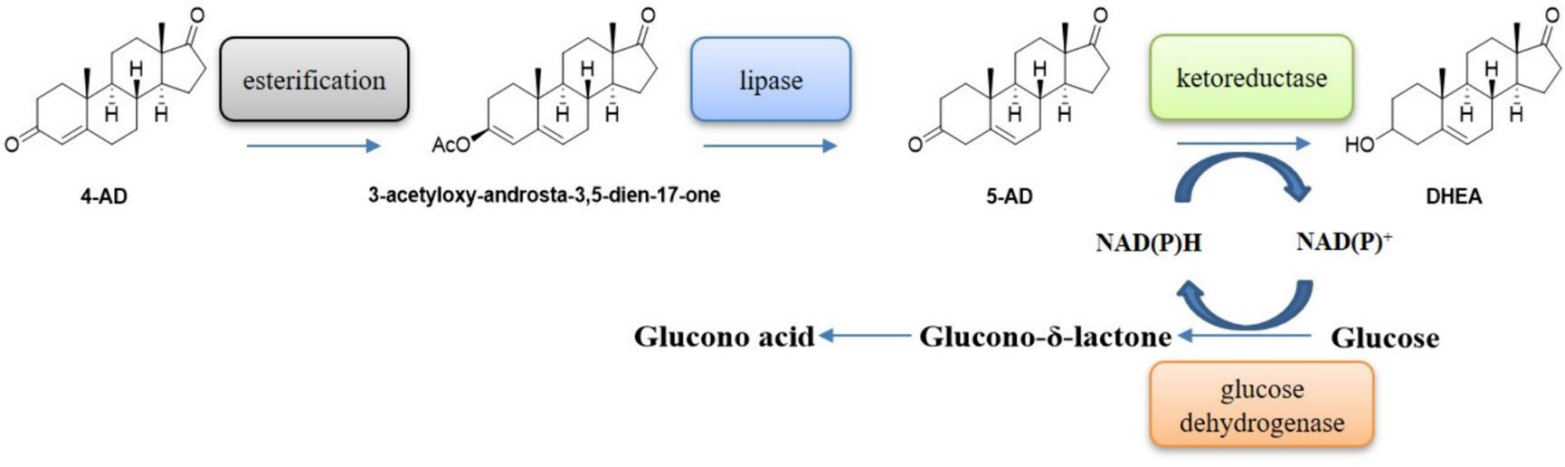
Chemical esterification coupled biocatalytic preparation of DHEA by 4-AD

We have attempted to further optimize the production of DHEA by chemical coupled biocatalysis (Scheme 2). Due to the shortcomings of the chemical isomerization of 4-AD, we have obtained the intermediate 5-AD from 4-AD by a milder and environmentally friendly esterification-hydrolysis strategy. The subsequent work on dual enzyme coupling reactions identified the sources of by-products in the production of DHEA with controlling strategies. We were able to achieve a conversion of 5-AD of 99.6% and a HPLC purity of 99.8% at 50 g/L of 3-ethoxy-androsta-3,5-dien-17-one. Further conversion of 5-AD to DHEA could achieve a by-product of 4-AD of 6.5% of total product and achieve a DHEA yield of 40.1 g/L. This study also broadens the multifunctional activity of SDRs and opened up new possibilities for the exploitation of enzyme-catalyzed reactions.

## Materials and methods

### Chemicals

3-Acetyloxy-androsta-3,5-dien-17-one and 5-AD were obtained from Aurisco Pharmaceutical Co. Ltd. (Taizhou, China). DHEA, 4-AD, and other chemicals were purchased from Sigma Chemical Reagent Co. Ltd. (Shanghai, China). His60 Ni Superflow Resin and Gravity Columns (TaKaRa Bio, Dalian, China) were purchased from TaKaRa Bio (Dalian, China). Thin-layer chromatography plate HSGF 254 was purchased from Jiangyou Silica Gel Open Co. Ltd. (Yantai, China). All chemicals were of analytical grade as commercially available.

### Synthesis of 3-acetyloxy-androsta-3,5-dien-17-one

Under nitrogen protection, 50 g of 4-AD, 50 g of toluene-p-sulfonic acid, and 200 mL of acetic anhydride were combined in the reactor and stirred at 40 °C until the complete reaction of the raw materials was confirmed by thin-layer chromatography (TLC). Subsequently, the reaction mixture was cooled to room temperature, and the product was precipitated by adding a substantial amount of water, filtered, and dried to yield 3-acetyloxy-androsta-3,5-dien-17-one.

### Identification of 3-acetyloxy-androsta-3,5-dien-17-one by LC−MS

LC−MS was performed using a SCIEX X500R Q-TOF MS system equipped with an electrospray ionization (ESI) source interface coupled with an ExionLC 30A UHPLC. Chromatographic separation was accomplished employing an ACQUITY UPLC HSS T3 column (100 Å, 1.8 μm, 2.1 mm × 100 mm) with a flow rate of 0.3 mL/min over a 12-min period. The mobile phases consisted of 0.1% (v/v) formic acid in water (A) and methanol (B) in an isocratic elution mode with a ratio of A:B = 1:9. MS was in positive-ion electrospray mode, and operating parameters were as follows: ion spray voltage, 5500 V; temperature, 500 °C; ion source gas, 50 psi; scan range, 80–800 Da; and MS collision energy, 10 eV.

### Screening of lipases to hydrolyze 3-acetyloxy-androsta-3,5-dien-17-one

The hydrolyzing 3-acetyloxy-androsta-3,5-dien-17-one capacity was assayed by using 0.1 g immobilized lipases and 20 g/L 3-acetyloxy-androsta-3,5-dien-17-one, added into 2 mL 0.2 M phosphate buffer saline (PBS) buffer (pH 6.0); the reaction mixture was incubated at 30 °C for 24 h. After the reaction, the supernatant was extracted by ethyl acetate for TLC analysis. Enzymes exhibiting hydrolytic activity were additionally screened by introducing 0.1 g of lipase into 2 mL of 0.2 M PBS at pH 6, supplemented with 50 g/L of 3-acetyloxy-androsta-3,5-dien-17-one. The reaction mixture was incubated at 30 °C for 8 h. Subsequently, the supernatant was extracted by ethyl acetate and substrate conversions were analyzed by HPLC.

### Optimization of lipase hydrolysis reaction

The product 5-AD generation rate was determined by measuring in the 1 mL assay mixture in different conditions for 2 h, composed of 25 g/L 3-acetyloxy-androsta-3,5-dien-17-one and 0.1 g Lipase PS Amano SD. The effect of pH was determined by measuring over the range from pH 4.0 to 10.0, the effect of temperature was determined in different temperatures from 20 to 50 °C, and the effect of organic solvent was determined by measuring in assay mixture 0.2 M PBS at pH 7 and 20% (v/v) different organic solvents. Following the reaction, the supernatant was extracted using ethyl acetate for subsequent HPLC analysis.

### Plasmids and strain construction

KR genes from five different sources, the GDH gene from *Bacillus subtilis*, and the alcohol dehydrogenase gene from *Lactobacillus brevis* (LbADH) were selected from the NCBI database (Table S1). The corresponding nucleotide sequences of these enzymes were codon-optimized, synthesized, and then inserted into the plasmid pET-28a (+), providing an N-terminal histidine-tag by Suzhou Genewiz Company. Then, the cloning vector transformed into competent *Escherichia coli* BL21(DE3) cells to obtain the recombinant *E. coli* strains.

### Expression and purification of the enzymes

All proteins with 6-histidine tag were purified following the same procedure (Chen et al. 2022). Recombinant *E. coli* BL21(DE3) cells were cultivated overnight at 37 °C and transferred to 50 mL LB medium with 50 μg/mL kanamycin. Then, the strain was cultured in a fermentation medium with a ratio of 1% (v/v) at 37 °C and 200 rpm. The fermentation medium contained 12 g/L yeast extract, 15 g/L peptone, 10 g/L glycerol, 8.9 g/L NaH_2_PO_4_·12H_2_O, 3.4 g/L KH_2_PO_4_, 2.67 g/L NH_4_Cl, 0.71 g/L Na_2_SO_4_, and 0.3 g/L MgSO_4_·7H_2_O. Upon reaching midlog phase (OD_600_ = 0.6–0.8), 1 M isopropyl β-D-1-thiogalactopyranoside (IPTG) was added into the expressed system, and the cultures were incubated at 24 °C and 200 rpm for an additional 18 h. Cells were collected by centrifugation at 8000 × *g* and 10 min at 4 °C. The harvested cells were then resuspended in 50 mM PBS at pH 6, followed by homogenization. Then, the target enzymes were purified with His60 Ni-Superflow Resin and Gravity Columns by nickel affinity chromatography according to the protocol instructions. The collected proteins were stored at −20 °C. Protein expression was analyzed by sodium dodecyl sulfate-polyacrylamide gel electrophoresis (SDS-PAGE), and protein concentration was measured by the Bradford method with the bovine serum albumin (BSA) as the standard.

### Reduction of 5-AD by dual enzyme coupling reaction in crude enzyme extracts catalysis form

Biocatalysis 5-AD by crude enzyme extract was investigated under the following conditions: 50 mL each of GDH/ADH and KR bacterium solution was harvested when OD_600_ was 10; the precipitate was collected by centrifugation and resuspended in PBS (pH 6.3) to 20 mL and then crushed as the crude enzyme. The 20-mL reaction mixture contained crude enzyme, 25 g/L 5-AD, 25 g/L glucose or 10% isopropanol (v/v), and 250 mg/L NAD^+^ and NADP^+^; then, the mixture continued stirring at 35 °C. The pH of the system was maintained at pH 6.3 by using the 1.5 M K_2_CO_3_ solution.

### Effect of catalytic substrate on the production of by-product 4-AD of the dual enzyme coupling reaction

The reaction mixture included 10 g/L 5-AD, 50 μL purified enzyme or 50 μL PBS as control, 450 μL 0.2 M PBS at pH 6, and different concentrations of catalytic substrates; the reaction mixture was incubated at 30 °C for 5h. After the reaction, the supernatant was extracted by ethyl acetate for HPLC analysis.

The characterization of NAD(P)^+^ and NAD(P)H influence on SwiKR isomerization products was performed with or without 500 mg/L NAD(P)^+^ or NAD(P)H. The characterization of NAD(P)^+^ and glucose influence on GDH isomerization products were performed with or without 50 g/L glucose and 500 mg/L NAD(P)^+^.

The effect of NAD(P)^+^ inhibition on GDH isomerization products was performed with 25 g/L glucose, 0–2 g/L NAD^+^, and NADP^+^. The effect of glucose inhibition on GDH isomerization products was performed with 500 mg/L NAD(P)^+^ and 0–400 g/L glucose.

### Analytical methods

The products were analyzed by TLC on a silica gel developed in the solvent system of ethyl acetate-n-hexane (1:2, v/v). After drying, the chromatograms detected by stained with 0.8 M potassium permanganate standard solution, followed by heating by a heat gun.

The 4-AD, DHEA, 5-AD, and 3-acetyloxy-androsta-3,5-dien-17-one concentration was analyzed by HPLC (Agilent Technologies 1260 Infinity II, USA) with an ELSD detector (Agilent Technologies G4260B 1260 Infinity II ELSD, USA) and an InertSustain C18 (5 μm) column (Shinjuku-ku, Tokyo, Japan) set at 50 °C. The HPLC conditions were as follows: The mobile phase was acetonitrile-methanol-water (25:35:40, v/v) from 0 to 16 min, linearly changed to acetonitrile-methanol (35:65, v/v) from 16 to 18 min, and maintained this mobile phase from 18 to 23 min. The pressure of the nebulizer gas (N2) was set at 4 bar, the temperature of the detector drift tube was 50 °C, and the injection volume was 10 μL.

### Structural analysis of catalytic products by NMR

After isolation, the structure of catalytic products was analyzed with the 1D NMR (^1^H and ^13^C) and 2D NMR (heteronuclear multiple-bond correlation (HMBC)) spectra. A Bruker Advance III 600 MHz spectrometer (Bruker Bio Spin Corp., Billerica, MA, USA) was applied, with chloroform-D1, and methanol-D4 as solvents. The ^1^H and ^13^C NMR spectra were recorded at 400 and 100 MHz, respectively.

## Results

### Synthesis of 3-ethoxy-androsta-3,5-dien-17-one and structural identification by LC/MS and NMR

3-Ethoxy-androsta-3,5-dien-17-one was obtained by esterifying the C3 hydroxyl group of 4-AD through toluene-p-sulfonic acid and acetic anhydride with the yield of 96.4% and purity 98.8% of HPLC. The results of LC/MS analysis of the sample are shown in Fig. S1, and the molecular ion peak of mass spectrometry results is consistent with the expected molecular weight size of the product (LC-MS (+) *m*/*z* 329.2091 [M+H] ^+^). More detailed structures were further determined by 1D NMR (^1^H and ^13^C) and 2D NMR (HMBC), and the results of spectra are shown in Figs. S4–6.

### Screening of substrate hydrolysis catalyzed by lipase

**Fig. 1:**
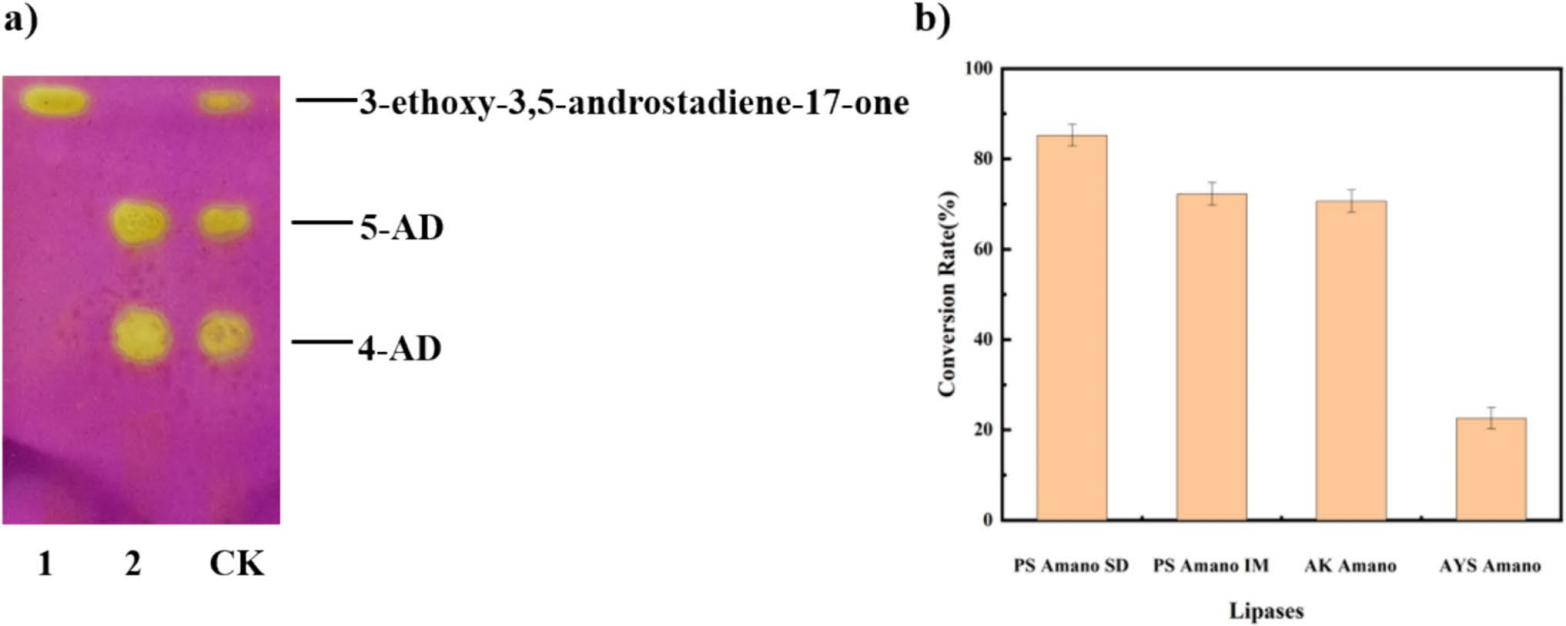
Screening of lipases with the capacity to hydrolyze 3-ethoxy-androsta-3,5-dien-17-one. **a** The TLC profile of the in vitro enzyme assay illustrates the hydrolytic activity of lipase. Lines 1 and 2 represent lipase samples without or with hydrolytic target substrate activity, respectively. CK (control) indicates standard compounds. Solvent system: ethyl acetate:n-hexane (1:2, v/v), chromogenic agent: 0.8 M potassium permanganate. **b** Comparison of substrate conversion rates of different lipases in second screening. The catalytic generation of 5-AD was assayed at pH 6, 30 °C for 8 h

For economic considerations in industrial applications and the convenience of experimentation, we have selected 11 kinds of commercially available and widely used lipases in our laboratory as candidate enzyme preparations for screening the hydrolysis of 3-ethoxy-androsta-3, 5-dien-17-one to produce 5-AD (Table S2). TLC was employed for the rapid detection of hydrolysis products. It was observed that four types of lipases produced 5-AD spots upon hydrolyzing the products. This indicates their catalytic activity towards substrate hydrolysis, including Lipase AYS Amano, Lipase PS Amano SD, Lipase PS Amano IM, and Lipase AK Amano. Subsequently, the four commercial lipases were re-screened with 50 g/L substrates, and the final conversion rates of the four lipases were compared after 8 h reaction. As depicted in Fig. 1 and Fig. S2, Lipase PS Amano SD exhibited the highest conversion rate at 85.2%; consequently, it was chosen for further investigation of catalytic conditions.

### Optimization of ester hydrolysis

**Fig. 2:**
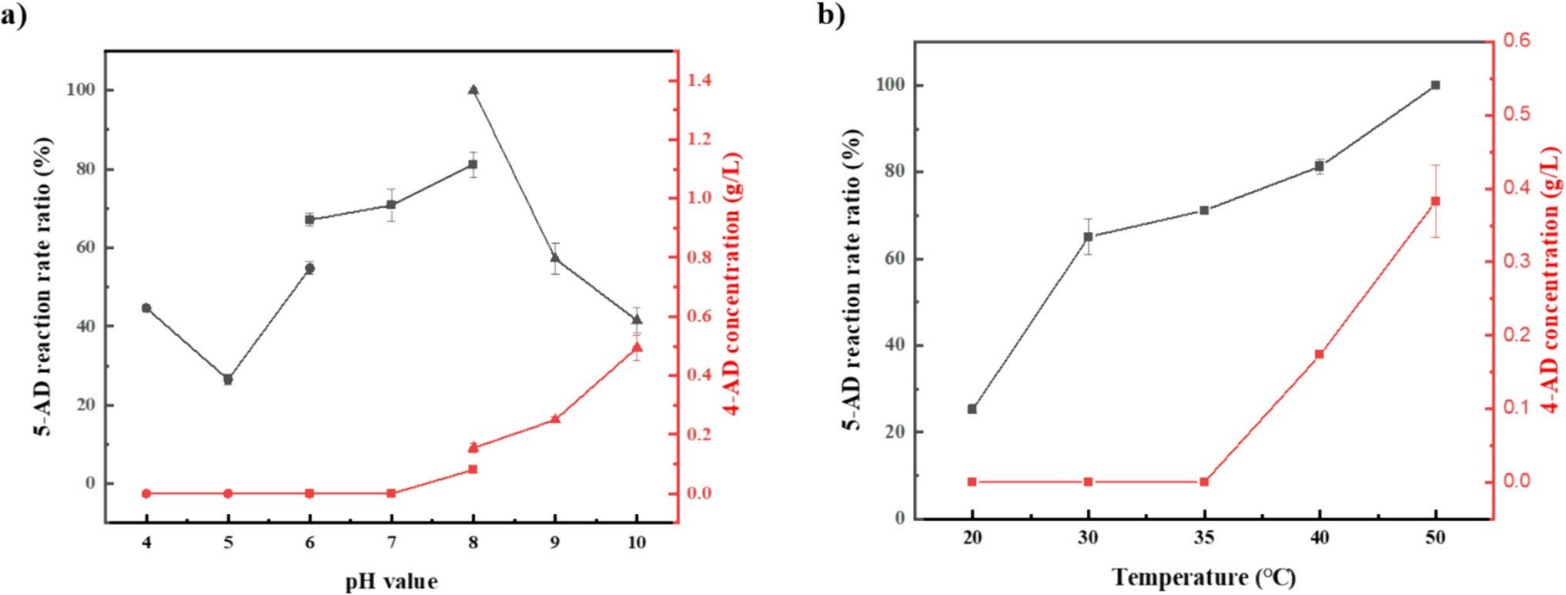
Optimization of reaction conditions of the hydrolysis reaction: **a** the reaction pH; **b** the reaction temperature. The buffer used in the assay mixture with the following: acetate (circles) (pH 4.0–6.0), phosphate (square) (pH 6.0–8.0), and Tris–HCl (triangles) (pH 8.0–10.0). The black line represents the relative reaction rate of the target product 5-AD, and the red line represents the yield of the by-product 4-AD. The maximum generation rate of 5-AD was set to 100%

We set 3-ethoxy-androsta-3,5-dien-17-one as the substrate and Lipase PS Amano SD as the catalyst while testing the following reaction conditions: temperature and pH values. As illustrated in Fig. 2, the optimum reaction condition for the highest reaction rate is 50 °C, pH 8.0 in Tris-HCl buffer solution. However, spontaneous isomerization of the hydrolysis product 5-AD generates the by-product 4-AD at temperatures above 35 °C and pH 7.0, with an increase in by-product formation at higher temperatures and pH levels. Consequently, we selected 35 °C and pH 7.0 as the catalytic reaction conditions.

**Fig. 3:**
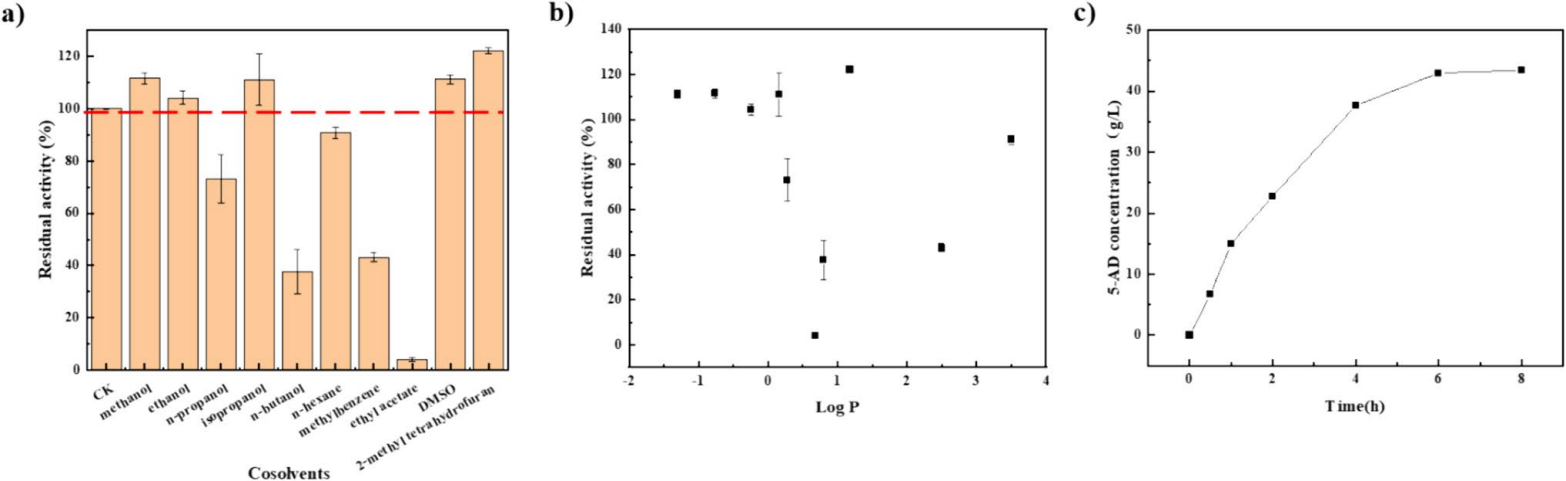
Organic solvent influence on the Lipase PS Amano SD hydrolysis activity. **a** Effect of different organic solvents on lipase activity. **b** Effect of logP value of organic solvent on lipase activity. **c** Process monitoring of Lipase PS Amano SD hydrolysis of 3-ethoxy-androsta-3,5-dien-17-one was assayed under optimal conditions for 8 h. The hydrolysis activity for the generation of 5-AD in the pure aqueous phase was set to 100%

Co-solvents were screened to improve the substrate solubility and catalytic efficiency. The following organic solvents were selected and categorized based on their log*P* values and the carbon chain length, respectively: methanol (−0.76), ethanol (−0.24), n-propanol (0.28), isopropanol (0.16), n-butanol (0.80), n-hexane (3.50), methylbenzene (2.50), ethyl acetate (0.68), DMSO (−1.30), and 2-methyl tetrahydrofuran (1.18). The co-solvent with the highest increase in catalytic efficiency was 2-methyl tetrahydrofuran, which increased the enzyme activity by 22.1% in comparison to the pure aqueous phase system (Fig. 3a). As shown in Fig. 3b, except for 2-methyl tetrahydrofuran, there was a significant correlation between log*P* and catalytic efficiency for the added co-solvents, in which catalytic efficiency showed a general trend of decreasing and then increasing with increasing log*P* values.

In summary, we implemented the optimization results by incorporating the addition of 20% (v/v) 2-methyl tetrahydrofuran into the aqueous system at 35 °C and pH 7.0 to catalyze 50 g/L of 3-acetyloxy-androsta-3,5-dien-17-one in a 50-mL system. As shown in Fig. 3c, 42.98 g/L 5-AD was obtained with a conversion of 99.6% and a HPLC purity of 99.8% after 6 h.

### Enzyme screening and catalytic results of dual enzyme coupling system

**Fig. 4:**
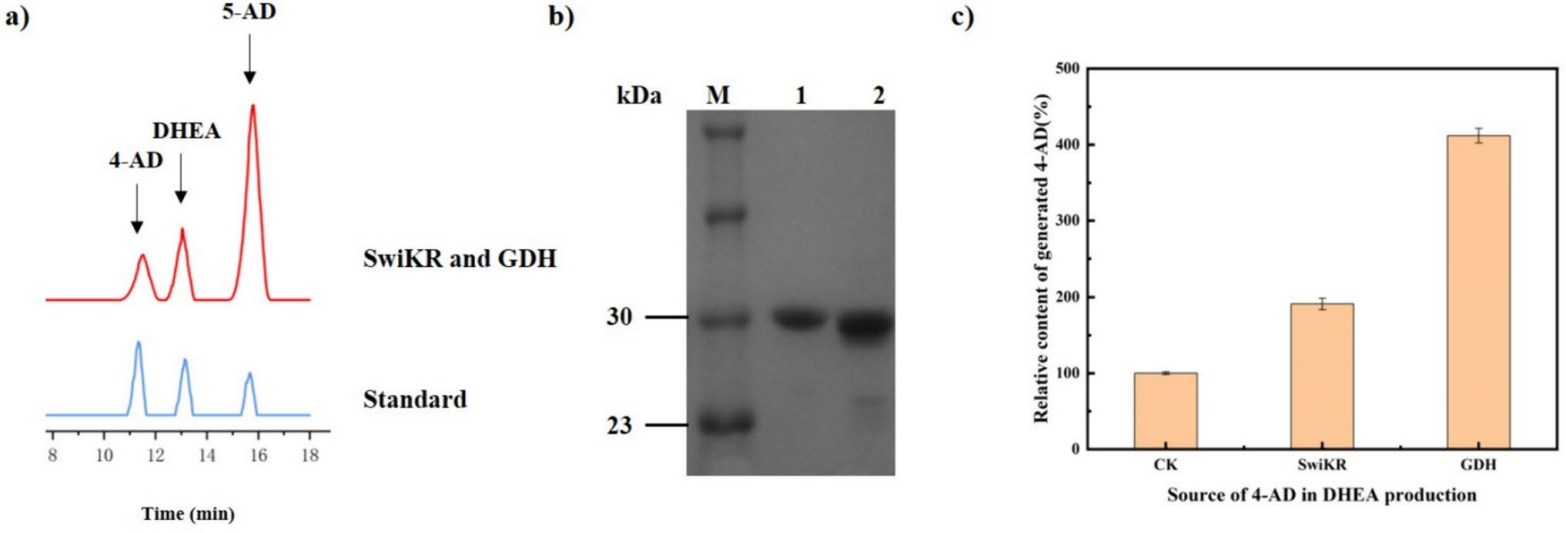
Results of a screening for the catalytic generation of DHEA by dual enzyme coupling system and sources of 4-AD generation. **a** The HPLC chromatography of reaction mixture composition of SwiKR and GDH and standard compounds was shown. The reaction was assayed at pH 6.3, 25 g/L 5-AD, 25 g/L glucose, 250 mg/L NAD^+^ and NADP^+^, at 35 °C. **b** Sodium dodecyl sulfate polyacrylamide gel electrophoresis (SDS−PAGE) analysis of SwiKR and GDH expression and purification. Lane M, marker; lane 1, purified GDH; lane 2, purified SwiKR; and loading volume, 5 μL. **c** The source of by-product 4-AD produced by each part of the catalytic system. SwiKR or GDH pure enzyme were used in the activity assay. The 4-AD content produced by the enzyme-free system was set as a control, and the amount of 4-AD produced by the remaining three parts was removed from the control

We successfully constructed and expressed five kinds of different sources of KRs and two kinds of coenzyme cycle enzymes. Whole-cell catalysis was employed to screen for the optimal combination of these enzymes, prioritizing high regioselectivity and stereoselectivity toward DHEA. KR from *Sphingomonas wittichii* (Anna et al. 2014) (SwiKR) and GDH from *Bacillus subtilis* were identified with relatively high activity (Fig. 4a; Figs. S7–8). It was noticed that the by-product 4-AD accumulated gradually over time during the catalytic process and comprised a large proportion of the total substrate amount (23.8%).

### Source of 4-AD in DHEA production

We purified SwiKR and GDH with a N-terminal His-tag by nickel column, the molecular size of the purified enzymes was characterized to be approximately 29.8 kDa and 30.2 kDa respectively based on SDS-PAGE, which is consistent with the predicted protein size (Fig. 4b). From the results of the isomerization reaction of the single substrate 5-AD catalyzed with or without pure enzyme (Fig. 4c; Figs. S9–10), both SwiKR and GDH had ketosteroid isomerase activity and were the main sources of by-products in the production of DHEA, and the GDH isomerization activity is 215.5% higher than that of SwiKR.

### Control of by-products in DHEA production

**Fig. 5:**
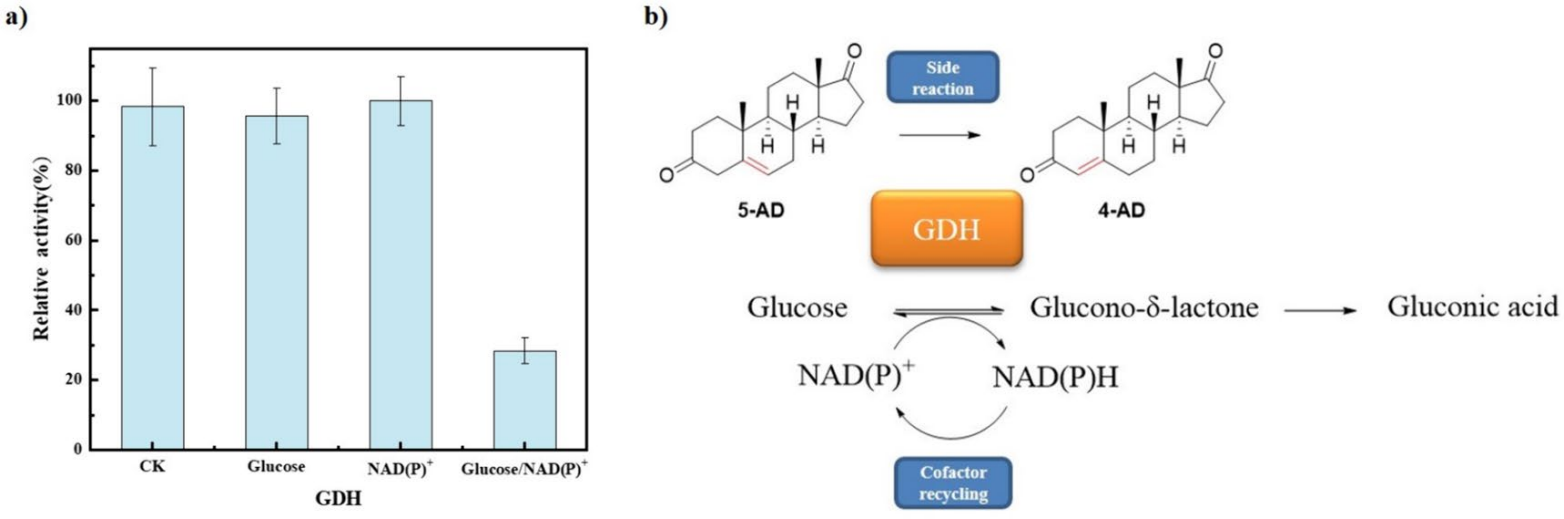
Factors influencing the isomerization activity of GDH enzyme and their catalytic reactions: **a** Effects of glucose and NAD(P)^+^ on the isomerization activity of GDH. **b** Primary/side catalytic reactions of GDH in dual enzyme coupling system. The control was the pure enzyme system without the addition of any other substrates. The reaction was assayed at pH 6.0, 10 g/L 5-AD, 50 g/L glucose, or 500 mg/L NAD^+^ and NADP^+^, at 30 °C

Thomas reported that NADH is key to the induction of time-dependent isomerization activity of human 3β-HSD/KSI (James L. Thomas et al. 1995); based on this, we aimed to investigate whether coenzyme factors and substrates of the coenzyme cycle also affect the isomerase activity of the promiscuous enzymes SwiKR and GDH. In contrast to the pure enzymatic reaction with a single substrate 5-AD, the other substrates of the dual enzyme coupling reaction, glucose and cofactors, had a significant influence to isomerase activity of SwiKR and GDH. As shown in Fig. 5a, the addition of glucose or NAD(P)^+^ alone barely affected the isomerase activity of GDH, but when added together, the two coenzyme cycle substrates significantly inhibited GDH isomerase activity by 71.1% compared to the control. On the other hand, as shown in Fig. 7a, the extra addition NAD(P)^+^ significantly promoted SwiKR isomerase activity by 106.8%, while the addition NAD(P)H increased SwiKR isomerase activity by 50.2%.

The inhibitory effect of glucose and NAD(P)^+^ on isomerization activity of GDH is probably due to the presence of two main catalytic reactions of GDH in systems, where 5-AD, glucose, and NAD(P)^+^ are present. One type of reaction catalyzes a coenzyme cycle that produces gluconate and NAD(P)H from the substrates glucose and NAD(P)^+^, and the other type of reaction catalyzes an isomerization reaction that produces 4-AD from the substrate 5-AD (Fig. 5b). In the absence of glucose and NAD(P)^+^ in the system, GDH mainly catalyzes the isomerization reaction; when the concentrations of glucose and NAD(P)^+^ are increased, GDH performs mainly coenzyme cycle activity and consequently the isomerization activity is inhibited.

**Fig. 6:**
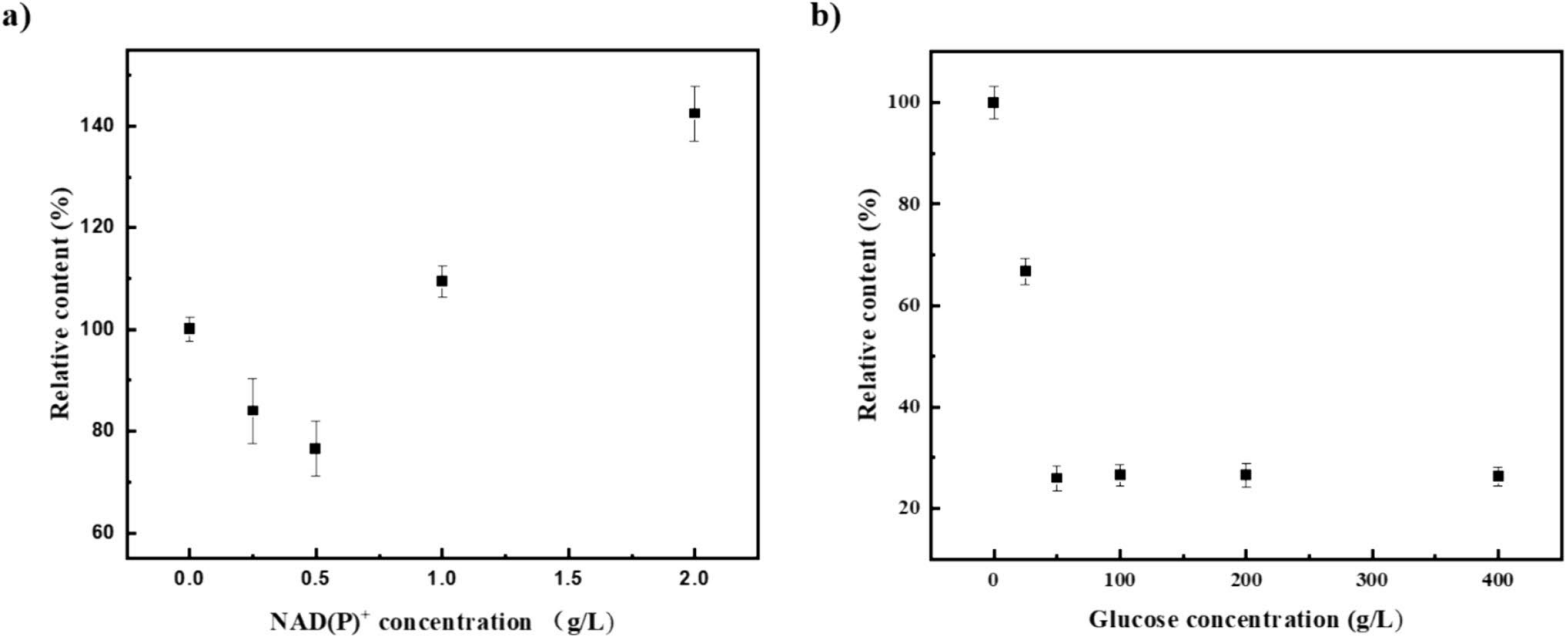
Effect of glucose and NAD(P)^+^ inhibition on GDH isomerization products: **a** The isomerization products of GDH pure enzyme were compared in the presence of 25 g/L glucose, 0–2.0 g/L NAD(P)^+^; **b** the isomerization products of GDH pure enzyme were compared in the presence of 500 mg/L NAD(P)^+^, 0–400 g/L glucose

To prove this hypothesis, we compared the inhibition of GDH isomerization activity by different concentrations of glucose and NAD(P)^+^ in the system. As shown in Fig. 6, in a system containing 25 g/L glucose, the GDH isomerization activity was lowest at 500 mg/L NAD(P)^+^ loading. Moreover, the addition of glucose to the catalytic system with 500 mg/L NAD(P)^+^ loading significantly inhibited the isomerization activity of GDH.

**Fig. 7:**
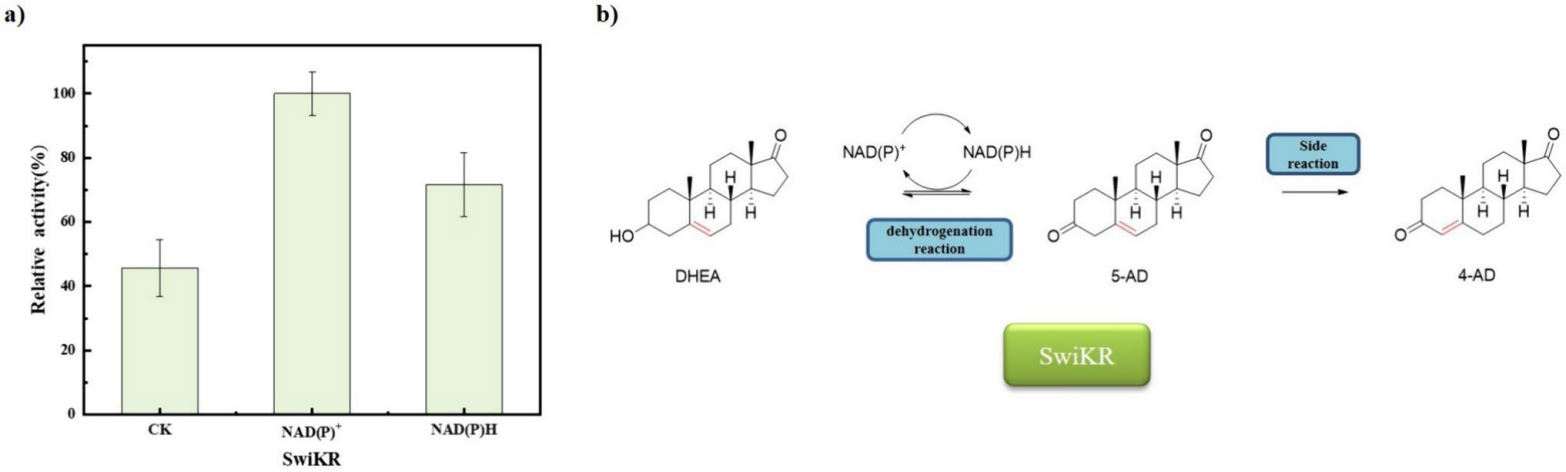
Factors influencing the isomerization activity of SwiKR enzyme and their catalytic reactions: **a** Effects of oxidation or reduction form cofactors on the isomerization activity of SwiKR; **b** primary/side catalytic reactions of SwiKR in dual enzyme coupling system. The control was the pure enzyme system without the addition of any other substrates. The reaction was assayed at pH 6.0, 10 g/L 5-AD, 50 g/L glucose, or 500 mg/L NAD^+^ and NADP^+^, at 30 °C

Similarly, the selective ketone reduction and ketosteroid isomerization scheme catalyzed by SwiKR are demonstrated in Fig. 7b. Since 5-AD is virtually insoluble in the aqueous phase, the content and composition of coenzymes become the key factor in controlling the reaction selectivity. Among the catalytic systems containing 5-AD and NAD(P)^+^, the reaction can only proceed in the direction of isomerization due to the absence of NAD(P)H. However, the addition of either oxidative or reductive forms of cofactors increased the isomerization activity of SwiKR (Fig. 7a). We investigated the effect of the oxidized or reduced form of the cofactor on the SwiKR isomerization activity is different, with the addition of NAD(P)^+^ enhancing the SwiKR isomerization activity 1.40 times more than NAD(P)H; thus, controlling the ratio of C_NAD(P)H_/C_NAD(P)+_ in the reaction system affects the production of SwiKR by-products. In addition, NAD(P)H is essential for the conversion of 5-AD to DHEA, and increasing the ratio also accelerates the reaction rate of DHEA.

**Fig. 8:**
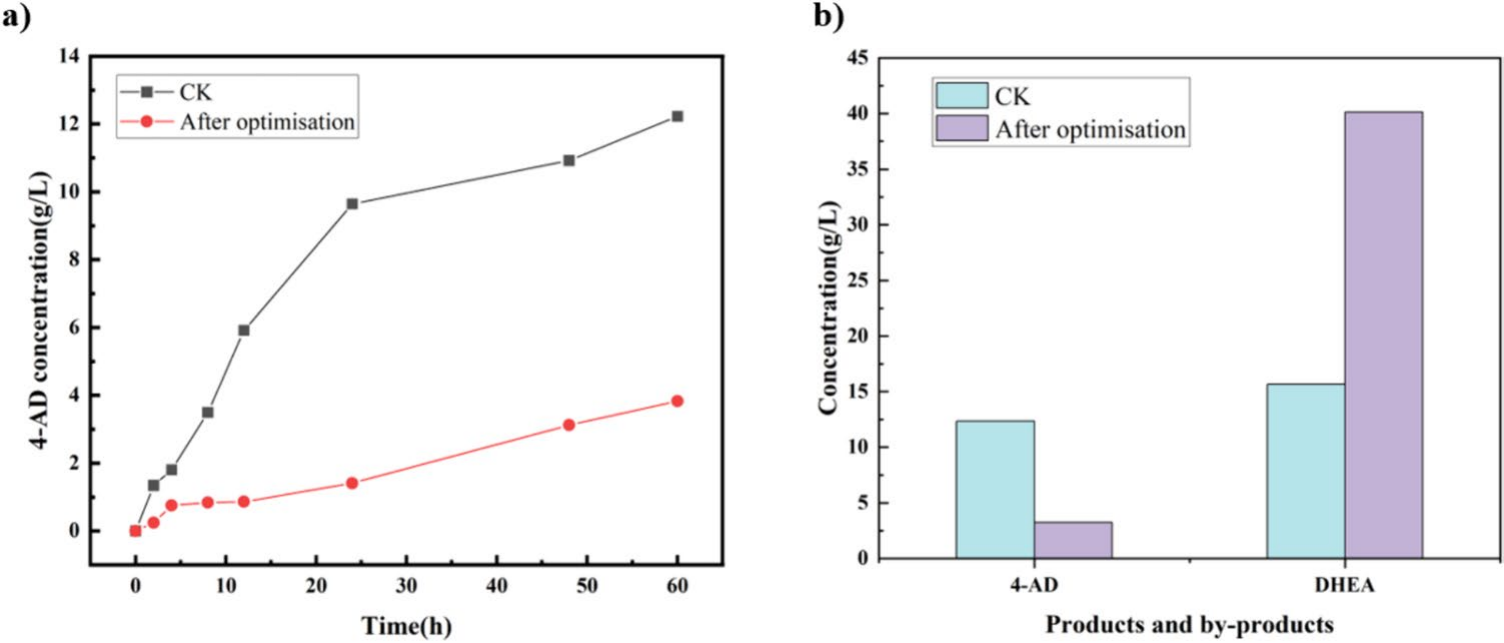
Effect of glucose on catalytic results: **a** Time course analysis of the production of 4-AD in the dual enzyme coupling system after optimization of glucose and cofactors. The catalytic process was monitored with or without 50 g/L glucose and 500 mg/L of NAD(P)^+^; **b** comparison of by-products containing products in the catalytic solution after the reaction

We assessed alterations in the production of the by-product 4-AD within the 5-AD system pre- and post-optimization of the SwiKR and GDH dual enzyme coupling system, incorporating coenzyme factors and glucose, as shown in Fig. 8. The results indicate that the presence of 50 g/L glucose and 500 mg/L of NAD(P)^+^ notably suppressed 4-AD production, which was reduced by an average of 75.3%, with the lowest concentration recorded at 14.6% compared to pre-optimized levels.

Based on the experimental results and theoretical analyses, we hypothesize that higher concentrations of glucose can effectively inhibit the GDH isomerization activity during the dual enzyme coupling process. Meanwhile, an increase in glucose concentration can stimulate the coenzyme cycle reaction, leading to an increase in the C_NAD(P)H_/C_NAD(P)+_ ratio that, in turn, inhibits SwiKR isomerization activity and escalates the rate of DHEA production.

## Discussion

The most facile synthetic scheme to obtain 5-AD reported in the literature currently is the isomerization via large amounts of tert-butanol and tert-butanol potassium to obtain the transition state, followed by quenching with acetic acid to obtain the precipitated 5-AD from the solution (Fryszkowska et al. 2016). The conversion of the 4-AD to the 5-AD involves deprotonation to form an intermediate complex (IS), and the calculated free energy profile for the reaction is shown in Fig. S3 (David et al. 1991; Fuller et al. 2019; Kedzierski et al. 2020). As IS has a higher energy level, the chemical isomerization process requires a large amount of OH^−^ to push the thermodynamic equilibrium from 4-AD to IS. The followed acid quenching allows the reprotonation of IS to 5-AD in a kinetics privilege (Pollack et al. 1986). However, due to the presence of OH^-^ at the beginning of acid quenching, significant amount of 4-AD can be formed and difficult to be separated in the process that followed. On the other hand, we proposed Scheme 2 to obtain 5-AD by chemical esterification of 4-AD followed by enzymatic hydrolysis by lipase, which obtains IS under acidic to neutral environment followed by its preferential formation to 5-AD. Thus, the proposed route is more environmentally friendly and has a higher catalytic efficiency and conversion rate.

We quickly screened a commercial lipase PS Amano SD as our enzymatic reagent using TLC and HPLC. Then, we optimized the reaction conditions such as temperature and pH to prevent the generation by-products during the production of 5-AD. Because 3-acetyloxy-androsta-3,5-dien-17-one is practically insoluble in water, these water-insoluble substrates are often catalyzed in organic solvents instead of aqueous reaction media, significantly enhancing the enzyme’s reaction rate (Klibanov 2001). Meanwhile, Lipase PS Amano SD belongs to *Burkholderia cepacia* lipase (BCL) that, like most lipases, has a phenomenon called interfacial activation to ensure a conformation more suitable for binding to the substrates (de Oliveira et al. 2017; Sánchez et al. 2018). As shown in Fig. 3b, most of the co-solvents miscible with water (log*P* < 0.16) showed high enzyme activity, with lower enzyme activity in solvents with log*P* between 0 and 2.5 and almost negligible activity at log*P* of 0.68 (4.0%), whereas the water-insoluble co-solvents showed a gradual increase in enzyme activity with increasing log*P*.

To the best of our knowledge, there are no studies on the source of by-products in the enzymatic conversion of 5-AD to DHEA. However, we found a large number of 4-AD production in our study, and both SwiKR and GDH were identified with ketosteroid isomerase activity. Under natural conditions, ketosteroid isomerase catalyzes the isomerization of ∆^5^-3-ketosteroid to ∆^4^-3-ketosteroid (Wu et al. 1997). This enzyme is one of the most active and efficient enzymes known which catalyzes the 5-AD reaction at a rate close to the diffusion-control limit (Pollack et al. 1986; Radzicka and Wolfenden 1995). Both SwiKR and GDH belong to the SDRs, which contain a large number of proteins (Wang et al. 2017) that can be distinguished to seven “classical” types of SDRs at a superficial level (Hult and Berglund 2007), and the members possess high functional diversity and very broad catalytic substrate spectrum.

It is widely recognized that a single protein of the SDRs catalyzes highly substrate-specific monofunctional reactions (Hoffmann and Maser 2008). However, there are some literatures on the ability of a few members of SDRs to accept more than their physiological substrate than previously thought. According to a series of studies by Thomas on human 3β-hydroxysteroid dehydrogenase/isomerase (3β-HSD/KSI), the enzyme contains both dehydrogenase and isomerase activities on a single enzyme protein (Pletnev et al. 2006; Thomas et al. 2010; Thomas et al. 1990). Conradt and Roth both reported that GDH from *Bacillus* species possesses iminium reductase activity in addition to dehydrogenase activity (Conradt et al. 2015; Roth et al. 2017). Pollack reported that 5-AD is susceptible to be converted to 4-AD in the presence of OH^−^, with determined equilibrium constants *K*_eq_ = 2400 (Pollack et al. 1989). On this basis, we speculated that the electron-rich environment in the catalytic center of SDRs can easily trigger the isomerization of 5-AD.

After verifying the isomerization activity of GDH and SwiKR, we discovered that maintaining at least 50 g/L of glucose in the catalytic system not only suppressed the isomerization activity of GDH but also increased the amount of NAD(P)H in the system, which effectively inhibits the isomerization activity of SwiKR. Meanwhile, it also increased the production and conversion rate of DHEA. Thus, when designing the DHEA-producing system using SDRs, it is necessary to consider whether the enzymes have promiscuous substrate specificity and develop appropriate strategies to control catalytic selectivity.

In summary, our study unveils a novel revelation: the emergence of 4-AD as a by-product in the course of DHEA biosynthesis. This discovery is rooted in the utilization of SwiKR and GDH, well-documented biocatalysts in DHEA synthesis. Notably, these enzymes exhibit promiscuity, demonstrating isomerase activity in addition to their established oxidoreductase function. The optimization of the catalytic process as well as the specific role of glucose was investigated and significantly reduced the production of 4-AD and increased the catalytic efficiency of DHEA. Moreover, chemical esterification as well as enzymatic hydrolysis strategies provide high selectivity and conversion of 5-AD, an essential intermediate in the biocatalysis of DHEA. This study provides a promising strategy for the industrial production of DHEA, furthermore providing a unique insight into the source of impurities in biocatalytic processes.

## Supplementary information

ESM 1 (PDF 918 kb)

## Data Availability

The data generated and analyzed in the present study is included in this published article.

## References

[CR1] Anna F, Siegfried QM, Chandra GSS, Reddy AS, Reddy PS, Reddy DNK, Upadhya T, Vilas D (2014) Process for the preparation of dehydroepiandrosterone and its intermediates

[CR2] Artini PG, Simi G, Ruggiero M, Pinelli S, Berardino OMD, Papini F, Papini S, Monteleone P, Cela V (2012) DHEA supplementation improves follicular microenviroment in poor responder patients. Gynecol Endocrinol 28(9):669–673. 10.3109/09513590.2012.705386 22835219 10.3109/09513590.2012.705386

[CR3] Chatterton RT (2022) Functions of dehydroepiandrosterone in relation to breast cancer. Steroids 179:108970–108977. 10.1016/j.steroids.2022.108970 35122788 10.1016/j.steroids.2022.108970

[CR4] Chen H, Liu Y, Ren X, Wang J, Zhu L, Lu Y, Chen X (2022) Engineering of cyclodextrin glycosyltransferase through a size/polarity guided triple-code strategy with enhanced α-glycosyl hesperidin synthesis ability. Appl Environ Microb 88(17):1–13. 10.1128/aem.01027-22 10.1128/aem.01027-22 PMC9469708 35950845

[CR5] Conradt D, Schätzle MA, Husain SM, Müller M (2015) Diversity in reduction with short-chain dehydrogenases: tetrahydroxynaphthalene reductase, trihydroxynaphthalene reductase, and glucose dehydrogenase. ChemCatChem 7(19):3116–3120. 10.1002/cctc.201500605

[CR6] David H, Eames TC, Pollack RM (1991) Energetics of 3-oxo-delta 5-steroid isomerase: source of the catalytic power of the enzyme. Biochemistry 30(45):10849–10858. 10.1021/bi00109a007 1932007 10.1021/bi00109a007

[CR7] de Oliveira IP, Jara GE, Martínez L (2017) Molecular mechanism of activation of *Burkholderia cepacia* lipase at aqueous-organic interfaces. Phys Chem Chem Phys 19(46):31499–31507. 10.1039/c7cp04466f 29160871 10.1039/c7cp04466f

[CR8] Flynn MA, Weaver-Osterholtz D, Sharpe-Timms KL, Allen S, Krause G (1999) Dehydroepiandrosterone replacement in aging humans. J Clin Endocr Metab 84:1527–1533. 10.1210/jcem.84.5.5672 10323374 10.1210/jcem.84.5.5672

[CR9] Fryszkowska A, Peterson J, Davies NL, Dewar C, Evans G, Bycroft M, Triggs N, Fleming T, Gorantla SSC, Hoge G, Quirmbach M, Timmanna U, Poreddy SR, Reddy DNK, Dahanukar V, Holt-Tiffin KE (2016) Development of a chemoenzymatic process for dehydroepiandrosterone acetate synthesis. Org Process Res Dev 20(8):1520–1528. 10.1021/acs.oprd.6b00215

[CR10] Fuller J, Wilson TR, Eberhart ME, Alexandrova AN (2019) Charge density in enzyme active site as a descriptor of electrostatic preorganization. J Chem Inf Model 59(5):2367–2373. 10.1021/acs.jcim.8b00958 30793899 10.1021/acs.jcim.8b00958

[CR11] Gajarawala SN, Wood TA, Stanton AP (2019) What is the role of dehydroepiandrosterone in gynecologic practice. JAAPA-J Am Acad Phys 32(12):11–12. 10.1097/01.JAA.0000604888.50734.64 10.1097/01.JAA.0000604888.50734.64 31770299

[CR12] Genazzani AD, Stomati M, Bernardi F, Pieri M, Rovati L, Genazzani AR (2003) Long-term low-dose dehydroepiandrosterone oral supplementation in early and late postmenopausal women modulates endocrine parameters and synthesis of neuroactive steroids. Fertil Steril 80:1495–1501. 10.1016/S0015-0282(03)02253-2 14667889 10.1016/j.fertnstert.2003.06.005

[CR13] group Tehabcc (2002) Endogenous sex hormones and breast cancer in postmenopausal women: reanalysis of nine prospective studies. J Natl Cancer I 94(8):606–616. 10.1093/jnci/94.8.606 10.1093/jnci/94.8.606 11959894

[CR14] Hoffmann F, Maser E (2008) Carbonyl reductases and pluripotent hydroxysteroid dehydrogenases of the short-chain dehydrogenase/reductase superfamily. Drug Metab Rev 39(1):87–144. 10.1080/03602530600969440 10.1080/03602530600969440 17364882

[CR15] Hosoda H, Fuicushima DK, Fishm AJ (1973) Convenient, high yield conversion of androst-5-ene-3β,17β-diol to dehydroisoandrosterone. J Org Chem 38(24):4209–4201. 10.1021/jo00963a028 4271499 10.1021/jo00963a028

[CR16] Huang K, Cai H, Wu L (2019) Potential of dehydroepiandrosterone in modulating osteoarthritis-related pain. Steroids 150:108433. 10.1016/j.steroids.2019.108433 31229511 10.1016/j.steroids.2019.108433

[CR17] Huang K, Wu L (2018) Dehydroepiandrosterone: molecular mechanisms and therapeutic implications in osteoarthritis. J Steroid Biochem 183:27–38. 10.1016/j.jsbmb.2018.05.004 10.1016/j.jsbmb.2018.05.004 29787833

[CR18] Hult K, Berglund P (2007) Enzyme promiscuity: mechanism and applications. Trends Biotechnol 25(5):231–238. 10.1016/j.tibtech.2007.03.002 17379338 10.1016/j.tibtech.2007.03.002

[CR19] Ihenacho U, Sriprasert I, Mack WJ, Hamilton AS, Unger JB, Press MF, Wu AH (2022) A systematic review and meta-analysis of smoking and circulating sex hormone levels among premenopausal women. Nicotine Tob Res 24(11):1705–1713. 10.1093/ntr/ntac066 35291014 10.1093/ntr/ntac066

[CR20] Jia X, Sun C, Tang O, Gorlov I, Nambi V, Virani SS, Villareal DT, Taffet GE, Yu B, Bressler J, Boerwinkle E, Windham BG, JAd L, Matsushita K, Selvin E, Michos ED, Hoogeveen RC, Ballantyne CM (2020) Plasma dehydroepiandrosterone sulfate and cardiovascular disease risk in older men and women. J Clin Endocrinol Metab 105(12):1–24. 10.1210/clinem/dgaa518 32785663 10.1210/clinem/dgaa518 PMC7526732

[CR21] Ke S, Wei Y, Shi L, Yang Q, Yang Z (2013) Synthesis of novel steroid derivatives derived from dehydroepiandrosterone as potential anticancer agents. Anti-Cancer Agent Me 13(8):1291–1298. 10.2174/18715206113139990323 10.2174/18715206113139990323 23547874

[CR22] Kedzierski P, Zaczkowska M, Sokalski WA (2020) Extreme catalytic power of ketosteroid isomerase related to the reversal of proton dislocations in hydrogen-bond network. J Phys Chem B 124(18):3661–3666. 10.1021/acs.jpcb.0c01489 32293890 10.1021/acs.jpcb.0c01489 PMC7467711

[CR23] Klibanov AM (2001) Improving enzymes by using them in organic solvents. Nature 409:241–246. 10.1038/35051719 11196652 10.1038/35051719

[CR24] Nippoldt TB, Nair KS (1998) Is there a case for DHEA replacement. J Clin Endocr Metab 12(3):507–520. 10.1016/s0950-351x(98)80286-3 10.1016/s0950-351x(98)80286-3 10332570

[CR25] Pan X, Wu X, Kaminga AC, Wen SW, Liu A (2019) Dehydroepiandrosterone and dehydroepiandrosterone sulfate in Alzheimer’s disease: a systematic review and meta-analysis. Front Aging Neurosci 11:61. 10.3389/fnagi.2019.00061 30983988 10.3389/fnagi.2019.00061 PMC6449476

[CR26] Pletnev VZ, Thomas JL, Rhaney FL, Holt LS, Scaccia LA, Umland TC, Duax WL (2006) Rational proteomics V: structure-based mutagenesis has revealed key residues responsible for substrate recognition and catalysis by the dehydrogenase and isomerase activities in human 3beta-hydroxysteroid dehydrogenase/isomerase type 1. J Steroid Biochem 101(1):50–60. 10.1016/j.jsbmb.2006.06.004 10.1016/j.jsbmb.2006.06.004 PMC1971842 16889958

[CR27] Pollack RM, Bantia S, Bounds PL, Koffman BM (1986) pH dependence of the kinetic parameters for 3-oxo-.DELTA.5-steroid isomerase. Substrate catalysis and inhibition by (3S)-spiro[5.alpha.-androstane-3,2’-oxiran]-17-one. Biochemistry 25:1905–1911 3707917 10.1021/bi00356a011

[CR28] Pollack RM, Zeng B, Mack JPG, Eldin S (1989) Determination of the microscopic rate constants for the base-catalyzed conjugation of 5-androstene-3,17-dione. J Am Chem Soc 111:6419–6423

[CR29] Prier CK, Kosjek B (2019) Recent preparative applications of redox enzymes. Curr Opin Chem Biol 49:105–112. 10.1016/j.cbpa.2018.11.011 30554005 10.1016/j.cbpa.2018.11.011

[CR30] Radzicka A, Wolfenden R (1995) A proficient enzyme. Science 267:90–93. 10.1126/science.7809611 7809611 10.1126/science.7809611

[CR31] Roth S, Präg A, Wechsler C, Marolt M, Ferlaino S, Lüdeke S, Sandon N, Wetzl D, Iding H, Wirz B, Müller M (2017) Extended catalytic scope of a well-known enzyme: Asymmetric reduction of iminium substrates by glucose dehydrogenase. ChemBioChem 18(17):1703–1706. 10.1002/cbic.201700261 28722796 10.1002/cbic.201700261

[CR32] Sahu P, Gidwani B, Dhongade HJ (2020) Pharmacological activities of dehydroepiandrosterone: a review. Steroids 153:108507. 10.1016/j.steroids.2019.108507 31586606 10.1016/j.steroids.2019.108507

[CR33] Sánchez DA, Tonetto GM, Ferreira ML (2018) *Burkholderia cepacia* lipase: a versatile catalyst in synthesis reactions. Biotechnol Bioeng 115(1):6–24. 10.1002/bit.26458 28941272 10.1002/bit.26458

[CR34] Thomas JL, Frieden C, Nash WE, Strickler RC (1995) An NADH-induced conformational change that mediates the sequential 3β-hydroxysteroid dehydrogenase/isomerase activities is supported by affinity labeling and the time-dependent activation of isomeras. J Biol Chem 270:21003–21008. 10.1074/jbc.270.36.21003 7673125 10.1074/jbc.270.36.21003

[CR35] Thomas JL, Mack VL, Sun J, Terrell JR, Bucholtz KM (2010) The functions of key residues in the inhibitor, substrate and cofactor sites of human 3β-hydroxysteroid dehydrogenase type 1 are validated by mutagenesis. J Steroid Biochem 120:192–199. 10.1016/j.jsbmb.2010.04.015 10.1016/j.jsbmb.2010.04.015 PMC2891085 20420909

[CR36] Thomas JL, Myers RP, Rosik LO, Strickler RC (1990) Affinity alkylation of human placental 3β-hydroxy-5-ene-steroid dehydrogenase and steroid 5→4-ene-isomerase by 2α-bromoacetoxyprogesterone: evidence for separate dehydrogenase and isomerase sites on one protein. J Steroid Biochem 36(1-2):117–123. 10.1016/0022-4731(90)90121-8 2362440 10.1016/0022-4731(90)90121-8

[CR37] Tomo S, Banerjee M, Karli S, Purohit P, Mitra P, Sharma P, Garg MK, Kumar B (2022) Assessment of DHEAS, cortisol, and DHEAS/cortisol ratio in patients with COVID-19: a pilot study. Hormones 21(3):515–518. 10.1007/s42000-022-00382-x 35804262 10.1007/s42000-022-00382-x PMC9266078

[CR38] Tomo S, Banerjee M, Sharma P, Garg M (2021) Does dehydroepiandrosterone sulfate have a role in COVID-19 prognosis and treatment. Endocr Regul 55(3):174–181. 10.2478/enr-2021-0019 34523302 10.2478/enr-2021-0019

[CR39] Wang H, Xiao D, Zhou C, Wang L, Wu L, Lu Y, Xiang Q, Zhao K, Li X, Ma M (2017) YLL056C from *Saccharomyces cerevisiae* encodes a novel protein with aldehyde reductase activity. Appl Microbiol Biotechnol 101(11):4507–4520. 10.1007/s00253-017-8209-5 28265724 10.1007/s00253-017-8209-5

[CR40] Wierman ME, Kiseljak-Vassiliades K (2022) Should dehydroepiandrosterone be administered to women. J Clin Endocrinol Metab 107(6):1679–1685. 10.1210/clinem/dgac130 35254428 10.1210/clinem/dgac130 PMC9113789

[CR41] Wu ZR, Ebrahimian S, Zawrotny ME, Thornburg LD, Perez-Alvarado GC, Brothers P, Pollack RM, Summers MF (1997) Solution structure of 3-oxo-△^5^-steroid isomerase. Science 276(5311):415–418. 10.1126/science.276.5311.415 9103200 10.1126/science.276.5311.415

[CR42] Zhang X, Huang Y, Xu N, Feng W, Qiao J, Liu M (2023) Low serum dehydroepiandrosterone levels are associated with diabetic retinopathy in patients with type 2 diabetes mellitus. J Diabetes Invest 14(5):675–685. 10.1111/jdi.13997 10.1111/jdi.13997 PMC10119925 36811237

[CR43] Zhao A, Zhang X, Li Y, Wang Z, Lv Y, Liu J, Alam A, Xiong W, Xu J (2021) *Mycolicibacterium* cell factory for the production of steroid-based drug intermediates. Biotechnol Adv 53:107860. 10.1016/j.biotechadv.2021.107860 34710554 10.1016/j.biotechadv.2021.107860

[CR44] Zhou P, Fang Y, Yao H, Li H, Wang G, Liu Y (2018) Efficient biotransformation of phytosterols to dehydroepiandrosterone by *Mycobacterium* sp. Appl Biochem Biotechnol 186(2):496–506. 10.1007/s12010-018-2739-x 29654468 10.1007/s12010-018-2739-x

