# Supplementary material for: Sources and control of impurity during one-pot enzymatic production of dehydroepiandrosterone
Source: Appl Microbiol Biotechnol. 2024 Jun 29;108:399. doi: 10.1007/s00253-024-13221-3 (PMC11217079; doi:10.1007/s00253-024-13221-3)
Supplement: Supplementary file 1 [file 253_2024_13221_MOESM1_ESM.pdf]

Applied Microbiology and Biotechnology

Sources and control of impurity during one-pot enzymatic production of dehydroepiandrosterone

Jiawei Dai[1,2], Zheyi Wu[1,2], Zebin Liu[1,2], Chen Li [1,2], Linjiang Zhu[1,2], Hanchi Chen[1,2] and Xiaolong Chen[1,2,*]
1 Institute of Fermentation Engineering, Zhejiang University of Technology, Hangzhou, 310014, China
2 College of Biotechnology and Bioengineering, Zhejiang University of Technology, Hangzhou, 310014, China

* Xiaolong Chen. E-mail: richard_chen@zjut.edu.cn

Supplementary information

Supplementary Tables

Table S1 Strains for catalytic and their sources.

Enzyme type	Source	Sequence number
KR	*Sphingomonas wittichii*	WP_012049478.1
	Sphingopyxis sp. LC81	WP_037511539.1
	Sphingomonas sanxanigenens	WP_025291566.1
	Williamsia sp. 1138	WP_084836793.1
	Burkholderia territorii	WP_060257435.1
GDH	*Bacillus subtilis*	WP_003246720.1
LbADH	*Lactobacillus brevis*	1NXQ_A

The amino acid sequences of the strains used in this paper were obtained from the NCBI database, and we codon-optimized the corresponding nucleotide sequences as follows:

Gene sequence of SwiKR

gcgcgcctggcgggcaaagtggccattatcagtggcgcggcgcaaggcatgggcgcggcgaccgcccgcctgtttgcggcggaaggcg
cgaaagtggtgattggcgatgtgctggatgaaaaaggccgcgcggtggcggcggaactgggcgcggatgtggcgctgtatcagcatctgg
atgtgcgcgaagaggaacagtgggcggcgattgtgaaagcggcggtggatcgctttggcaaactggatattctggtgaacaacgcggcggt
gacccattttggcgcgagcgaagaactgcgcaaagaagatgcggaacgcgtgctgggcattaacctgattggcaccatgatgggcgtgaaa
catgcggtgccggcgctgaaagcgaacggccgcggcgtgattgtgaacattagcagcgtggatggcctgcgcggctgcaacggcctggtg
gcgtataccgcgagcaaatgggcggtgcgcggcattaccaaaagctatgcgtatgaatttggcccgctgggcattcgcgtggtgagcattcat
ccgggcggcgtgaacaccgaaatgggcaacccgggccatgaaagcgtggaaaccgtgaacgcgcgcagctttggccgcgtgccgctgc
agcgcattggcgaaccggaagaaattgcgcgcgcgaccctgtttgtgtgcagcgatgaagcgagctatatcagtggtgcggaaattgcggtg
gatggcggctggaccgcgggccattatgaaccggcgctgccgggctgcccggatcatctgcgcggctaa

Gene sequence of SphKR

cgcgcctggccggcaaagtggcgattgtgaccggcgcggcgcaaggcatgggcgcggcgaccgcgcgcctgtttgcggcggaaggcg
cgaaagtggtgctgtgcgatgtgattgaagataaaggccaagaaaccgcgcgcgaaattggcgaaagcgcggtgtttcagaaactggatgt
gcgcagcgaagcggattgggaacgcgtggtggcggaaaccgtgcagcgctttggcaaactggatattctgattaacaacgcggcgattgtg
cattttagcccgattgaagcgatgccggcggaagatattgaacgcgtgctggcgattaacgtgatgggcaccatgctgggcgcgaaatatgc
ggcgcgcgcgatgaccgaagcgggccgcggcgtgattgtgaacattagcagcgtggatggcctgcgcggctgcaacggcctgagcgcgt
ataccgcgagcaaatgggcggtgcgcggcctgagcaaaagcctggcgtatgaactgggcccgcgcggcattcgcgtgtgcaccgtgcatc
cgggcggcgtgaacacgcagatgggcaacccgaccggcctgaccggcgatgcgctgaacgtgggctatgaacgcgtgccgctgcagcg
cattggcgaaccggaagaaattgcgcgcgcgagcctgtttattgcgagcgatgatgcgagctatattagcggcgcggaactggcggtggat
ggcggctggagcgcgggctattatcagccgtttctgccgggcgcgccggcgagcctgatgggtcagccgtaa

Gene sequence of SsaKR

gccgcctggcgggcaaagtggcgattattagcggtgcggcgcaaggcatgggcgcggcgaccgcgcgcctgtttgcgagcgaaggcgc
gaaagtggtgctgggcgatgtgctggaagaaaaaggccgcgtggtggcggcggaaattggcgatgcggcgagctttcagaaactggatgt

gcgcgaagaagcggattgggcggcgattgtggcgcatgcgggcgaacgctttggcaaactggatattctggtgaacaacgcggcggtgac
ccattttggcgcggcggaagaactgcgcaaagcggatgtggaacgcgtgctgggcattaacctgattggcaccatgatgggcgtgaaacat
gcggtgccggcgctgaaagcgaacggcaaaggcgtgattgtgaacattagcagcgtggatggcctgcgcggctgcaacggcctggtggc
gtataccgcgagcaaatgggcggtgcgcggcattagcaaaagctatgcgtatgaatttggcccgtttggcattcgcgtggtgagcattcatcc
gggcggcgtgaacaccgaaatgggcaacccggcgggcgaagcggcggaagcggtgaacgcgcgccattttcgccgcgtgccgctgca
gcgcattggcgaaccggaagaaattgcgcgcgcgaccctgtttgtgtgcagcgatgaagcgagctatattagcggtgcggaaattgcggtg
gatggcggctggaccgcgggccattatgaaccggcgctgccgggctgcgcgccgagcctgctggcgtaag

Gene sequence of WilKR

gccgcctggaaggcaaaaccgcgattgtgaccggcggcgcgcaaggcatgggcagcgcgaccgtgcgcgtgatggtggaagaaggcg
cgaaagtggtgattgcggatctggcggaacaagcgggcaaaagcctggcggcggaactgggcgatgcggcgagcttttgccgcctggatg
tgagcagcgaaagcgattggcagaaagtgctggcgcataccctggaagtgcatggcaccgtgaacgtgctggtgaacaacgcgggcattc
agtattttgtgggcgtggaagatattgaagcggaacgcgtgatgcgcctgtttagcattaacgtgctgggcagcatgctgggcgtgaaaaccg
tggcgccgattatgaaaaaagcgggcgcgggcgtggtgattaacattagcagcctggatggctttcgcggcaccaacggcatgagcccgta
tgtggcgagcaaatgggcggtgcgcggcctgaccaaagcgcaagcgctggaactgggcccggtgattcgcgtggtgagcgtgcatccgg
gcggcgtgaacaccccgatgggcaacccgaccggcgacaccggcgaagccctgaacgcgccgtatggccgcgtgccgctgcgccgcat
tggcgaaccgattgaagtggcgcgcgtgaccgcgtttatggcgagcgatgatgcgagctatgtgagcggcagcgaaattgcggtggatggc
ggctggaccgcgggccattatcatgtgggcctgccgggcggcccggaagcgtaa

Gene sequence of BteKR

cgcgcctggaaggcaaagtggcgattgttaccggcgcggcgcaaggcatgggcgcggcgaccgcgcgcctgtttgtgcatgaaggcgcg
cgcgtggtgctgggcgatgtgctggaagaaaaaggccgcgcgctggcggcggaactgggcgatgcggcgatttttaccccgctggatgtg
agcgatgaaagcagctgggaaagcgcggtggcggtggcggtggatcgctttggcggcctggatattctggtgaacaacgcgggcgtgatg
cattgggcgccgattgaagatctggatgtggcgcgcaccgaacgcctgctggatattaacgtgctgggcaacctgctgggcgcgaaagcgg
tggtgccgaccatgaaaaaagcgggccgcggcgtgattgtgaacattagcagcgtggatggcctgcgcggcgtgaacggcctggcggcgt
ataccgcgagcaaatgggcggtgcgcggcctgaccaaagcgctggcgtatgaactgggcccggcgggcattcgtgtgtgtagcgttcatcc
gggcggcgttgataccaccctgggcaacccgggcggcctggtgggcgatgatctgcagagcaaatatgtgggcgtgccgctgcagcgcat
tggcgaaagcgaagatattgcgcgcgcgaccctgtttgtggcgagcgatgaagcgagctatattagcggcgcggaactggcggtggatgg
cggctggagcgcgggcacctattatccgggcctgccgggcaccccgccggcgctgatgccgaactaa

Gene sequence of GDH

tatccggatttaaaaggaaaagtcgtcgctattacaggagctgcttcagggctcggaaaggcgatggccattcgcttcggcaaggagcaggc
aaaagtggttatcaactattatagtaataaacaagatccgaacgaggtaaaagaagaggtcatcaaggcgggcggtgaagctgttgtcgtcca
aggagatgtcacgaaagaggaagatgtaaaaaatatcgtgcaaacggcaattaaggagttcggcacactcgatattatgattaataatgccgg
tcttgaaaatcctgtgccatctcacgaaatgccgctcaaggattgggataaagtcatcggcacgaacttaacgggtgcctttttaggaagccgtg
aagcgattaaatatttcgtagaaaacgatatcaagggaaatgtcattaacatgtccagtgtgcacgaagtgattccttggccgttatttgtccacta
tgcggcaagtaaaggcgggataaagctgatgacagaaacattagcgttggaatacgcgccgaagggcattcgcgtcaataatattgggcca
ggtgcgatcaacacgccaatcaatgctgaaaaattcgctgaccctaaacagaaagctgatgtagaaagcatgattccaatgggatatatcggc
gaaccggaggagatcgccgcagtagcagcctggcttgcttcgaaggaagccagctacgtcacaggcatcacgttattcgcggacggcggt
atgacacaatatccttcattccaggcaggccgcggttaa

Gene sequence of LbADH

agcaaccgcctggatggcaaagtggcgattattaccggcggcaccctgggcattggcctggcgattgcaaccaaatttgtggaagaaggcg
cgaaagtgatgattaccggccgccatagcgatgtgggcgaaaaagcggcaaaaagcgtgggcacccctgatcagattcagtttttcagcat

gatagcagcgatgaggatggctggaccaaactgtttgatgcgaccgaaaaagcgtttggcccggtgagcaccctggtgaataatgcgggtat
tgcggtgaacaaaagcgtggaagaaaccaccaccgccgaatggcgcaaactgctggcagttaacttagatggcgtgtttttggcacccgcc
tgggcattcagcgcatgaaaaataaaggcctgggcgcgagcattattaacatgagcagcattgaaggctttgtgggcgatccgagcctgggc
gcgtataatgcgagcaaaggcgcggttcgcattatgagcaaaagcgcggcgttagattgcgcgctgaaagattatgatgtgcgcgtgaacac
cgtgcatccgggctatattaaaaccccgctggtggatgatctgccgggcgcggaagaagcgatgagccaacgtaccaaaaccccgatgggt
catattggcgaaccgaacgatattgcgtatatttgcgtgtatctggcgagcaacgaaagcaaatttgcgaccggcagcgaatttgtggtggatg
gcggttataccgcgcagtaa

Table S2 TLC for preliminary screening of lipases with 3-ethoxy-androsta-3, 5-dien-17-one hydrolyzing activity.

Enzyme type	Source	Hydrolytic substrate activity
Novozym 435	*Candida antarctica*	NA
Novozym 40086	*Rhizomucor miehei*	NA
Lipozym TL IM	*Thermomyces lanuginosus*	NA
Lipozym RM IM	*Rhizomucor miehei*	NA
Lipase AYS Amano	*Candida rugosa*	+
Lipase AK Amano	*Pseudomonas fluorescens*	++
Lipase AS Amano	*Aspergillus Niger*	NA
Lipase PS Amano IM	*Pseudomonas ceponiae*	++
Lipase PS Amano SD	*Pseudomonas ceponiae*	+++
CALB	*Candida Antarctic*	NA
CRL	*Candida rugosa*	NA

The amount of + represents the spot size of 5-AD after catalytic reaction, NA=No activity, indicating no catalytic activity.

Supplementary Figures

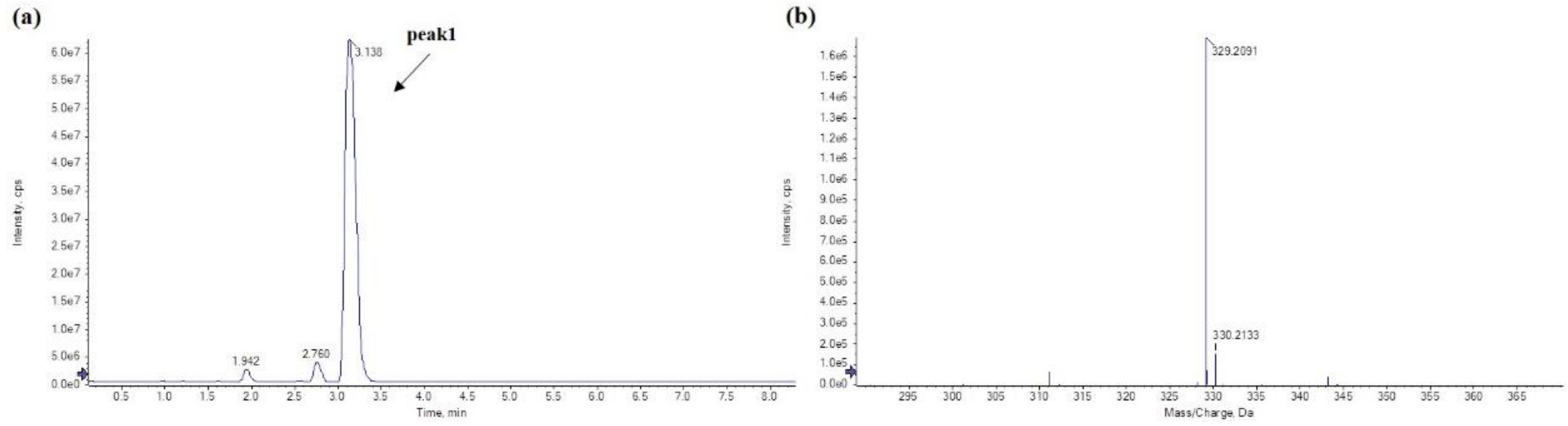

Figure. S1 Substrate analysis spectra of 3-acetyloxy-androsta-3, 5-dien-17-one: (a) TIC spectra; (b) positive ESI-MS of the specific product peaks 1 in (a).

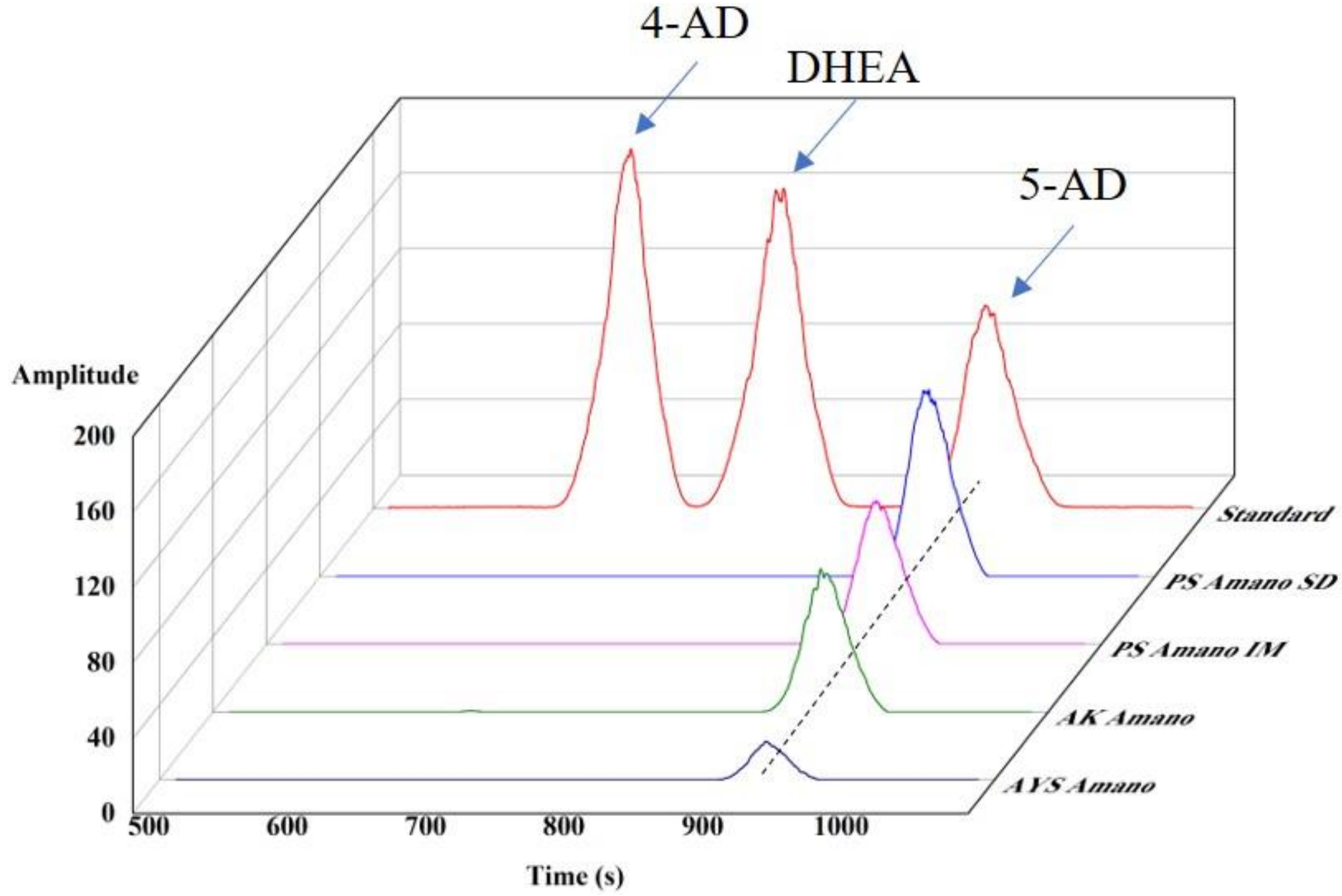

Figure. S2 The HPLC chromatography of reaction mixture of different lipases after 8 h at pH 6, 30 °C, compared with standard compounds.

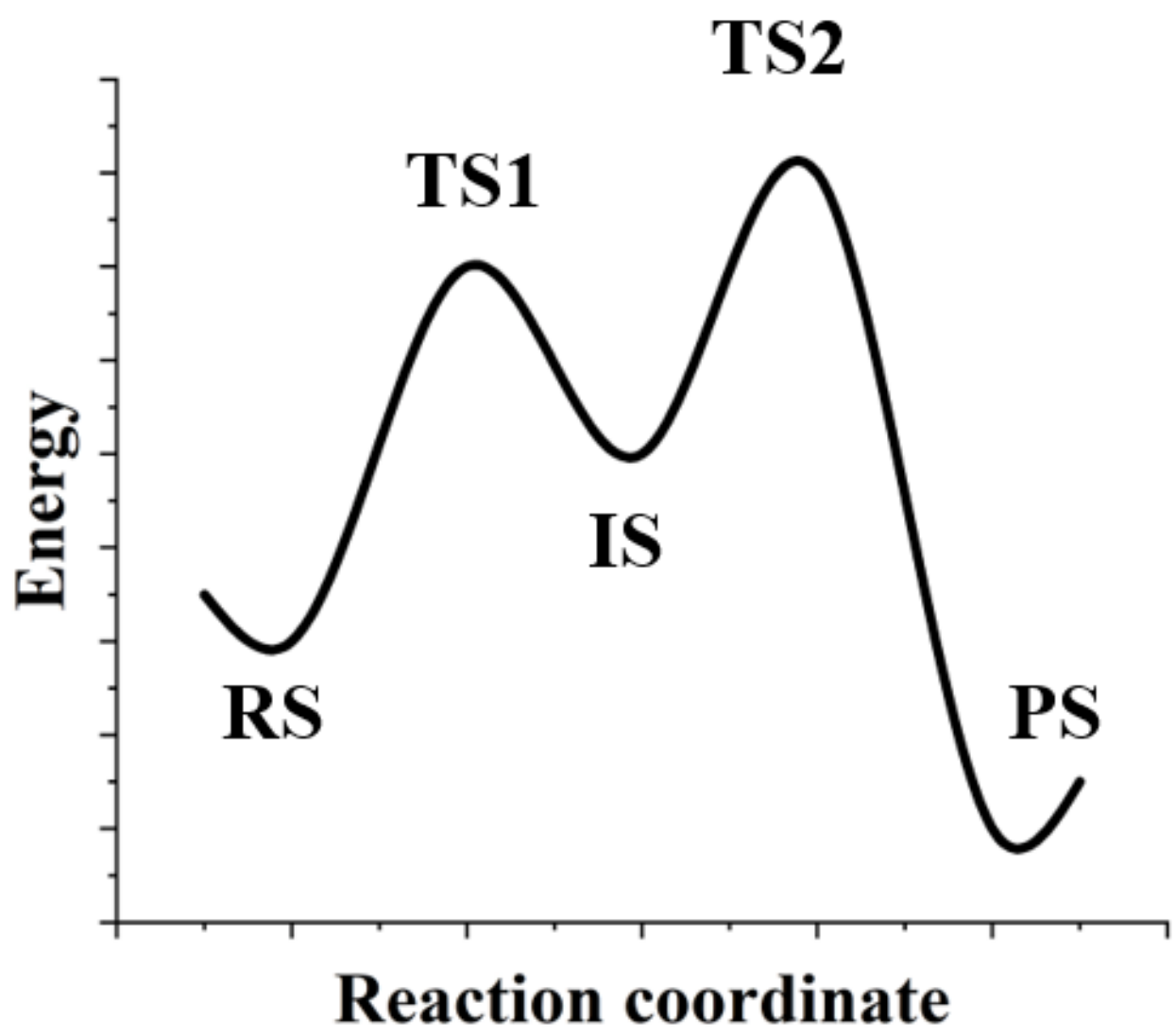

Figure. S3 Free energy profile for the reaction of the 5-AD (substrate, RS) to the 4-AD (product, PS)

Analytical data for catalytic products

Lipase substrate synthesis: 3-acetyloxy-androsta-3,5-dien-17-one

^{1}H NMR (400 MHz, $CDCl_3$) δ 5.68 (s, 1H), 5.47 (s, 1H), 5.39 (s, 1H), 2.51 – 0.79 (m, 45H).

^{13}C NMR (101 MHz, $CDCl_3$) δ 169.18, 168.69, 159.39, 146.82, 139.48, 123.32, 116.74, 111.05, 54.02, 48.33, 44.57, 34.93, 33.45, 33.10, 30.63, 29.73, 28.69, 24.55, 20.95, 20.37, 18.56, 15.26.

SwiKR catalyzes the conversion of 5-androstene-3,17-dione into dehydroepiandrosterone

^{1}H NMR (400 MHz, CH_3DO) δ 4.87 (s, 1H), 3.47 – 3.32 (m, 1H), 2.47 (dd, *J* = 19.1, 8.8 Hz, 1H), 2.31 – 0.89 (m, 18H).

^{13}C NMR (101 MHz, CH_3DO) δ 142.46, 121.90, 72.29, 51.82, 42.99, 38.47, 37.83, 36.69, 32.79, 32.69, 32.26, 31.86, 22.81, 21.48, 19.88, 13.94.

GDH and SwiKR catalyzes the conversion of 5-androstene-3,17-dione into 4-androstene-3,17-dione

^{1}H NMR (400 MHz, CH_3DO) δ 4.87 (s, 1H), 3.98 (s, 1H), 1.80 – 0.07 (m, 31H).

^{13}C NMR (101 MHz, CH_3DO) δ 202.09, 174.47, 124.33, 55.29, 52.06, 40.03, 36.72, 36.61, 36.28, 34.69, 33.68, 32.52, 32.03, 22.64, 21.38, 17.66, 14.09.

NMR Spectra

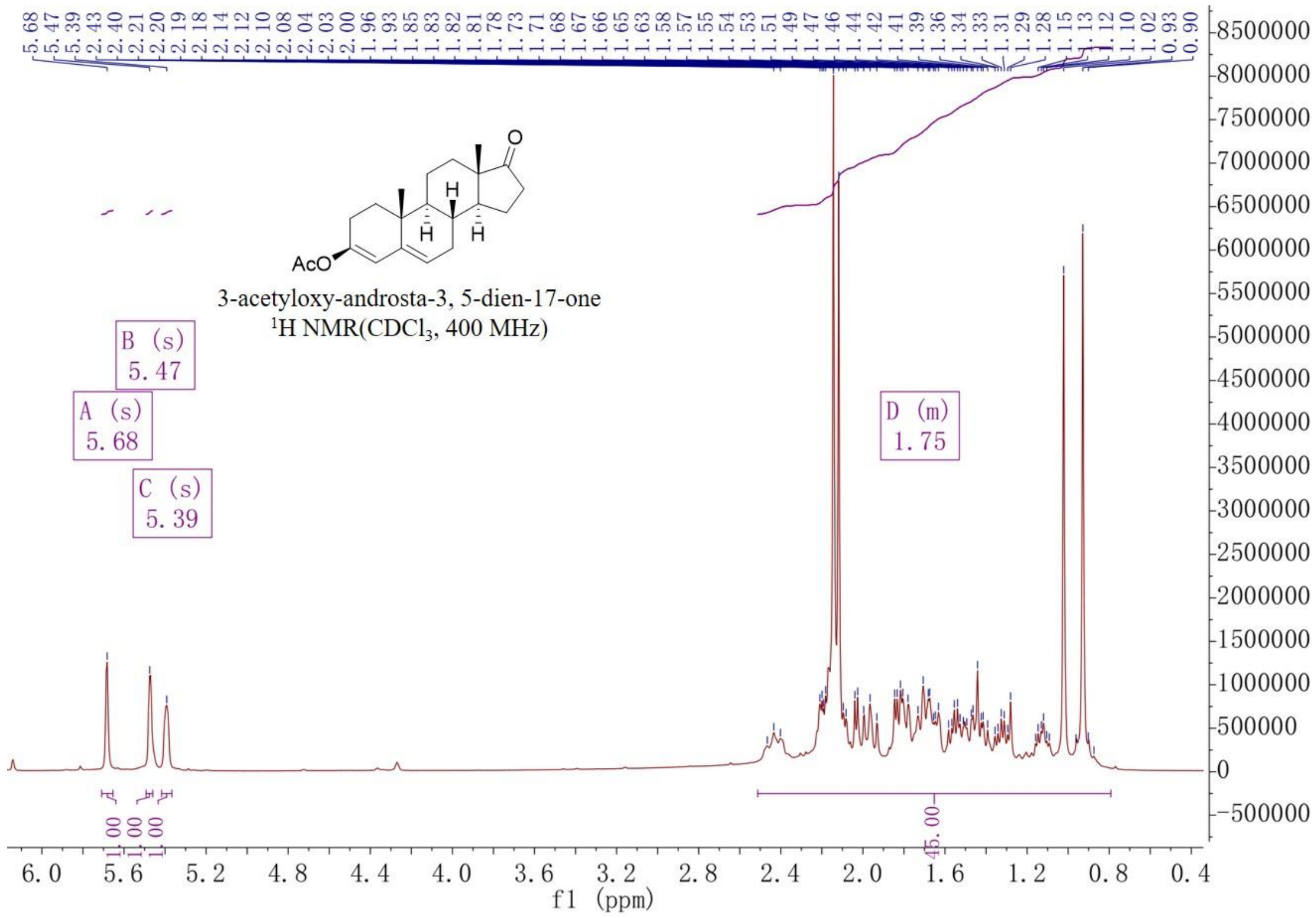

Figure. S4 ^{1}HNMR spectrum of 3-acetyloxy-androsta-3,5-dien-17-one in DMSO-d6 (400 MHz).

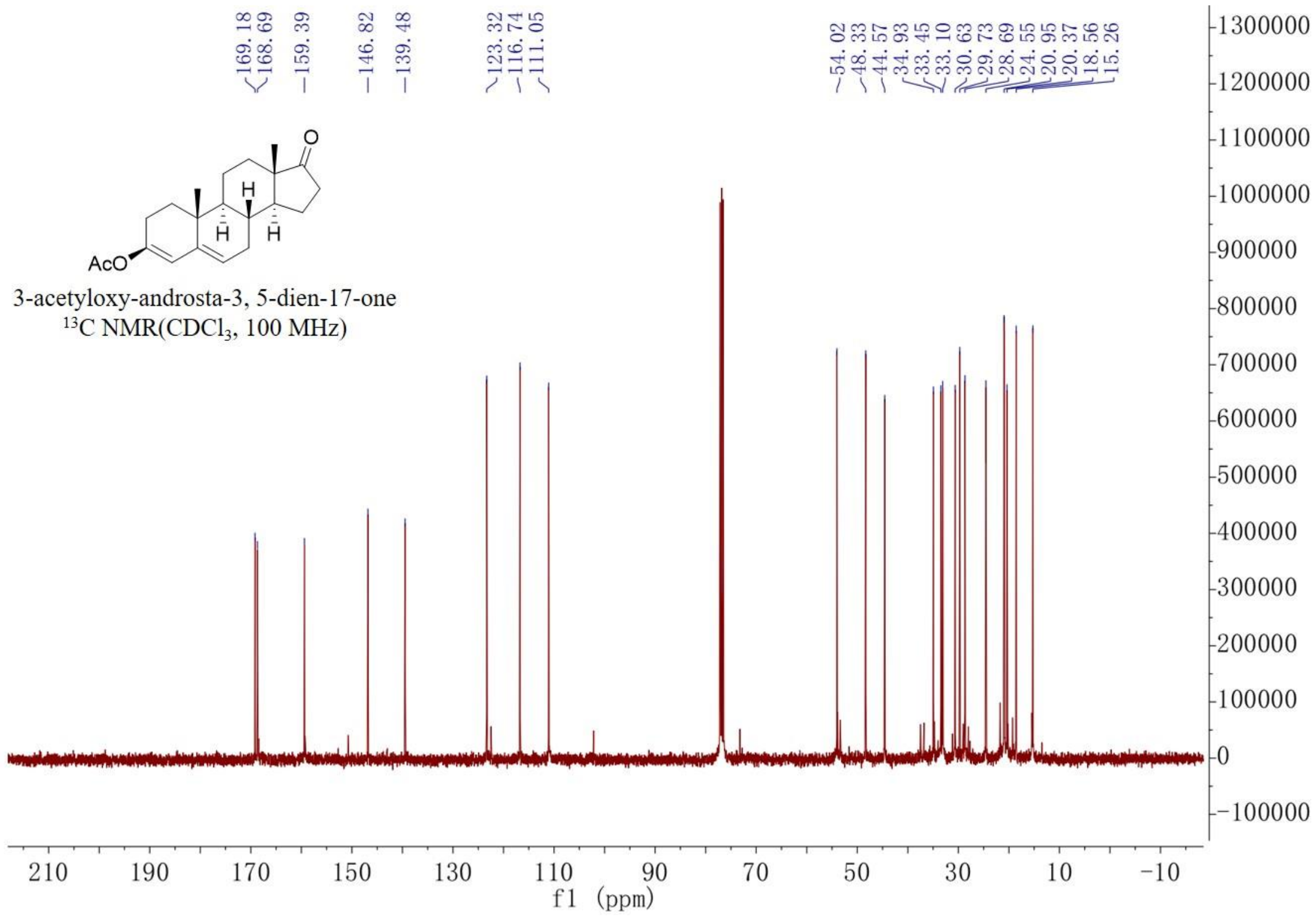

Figure. S5 ^{13}CNMR spectrum of 3-acetyloxy-androsta-3,5-dien-17-one in DMSO-d6 (100 MHz).

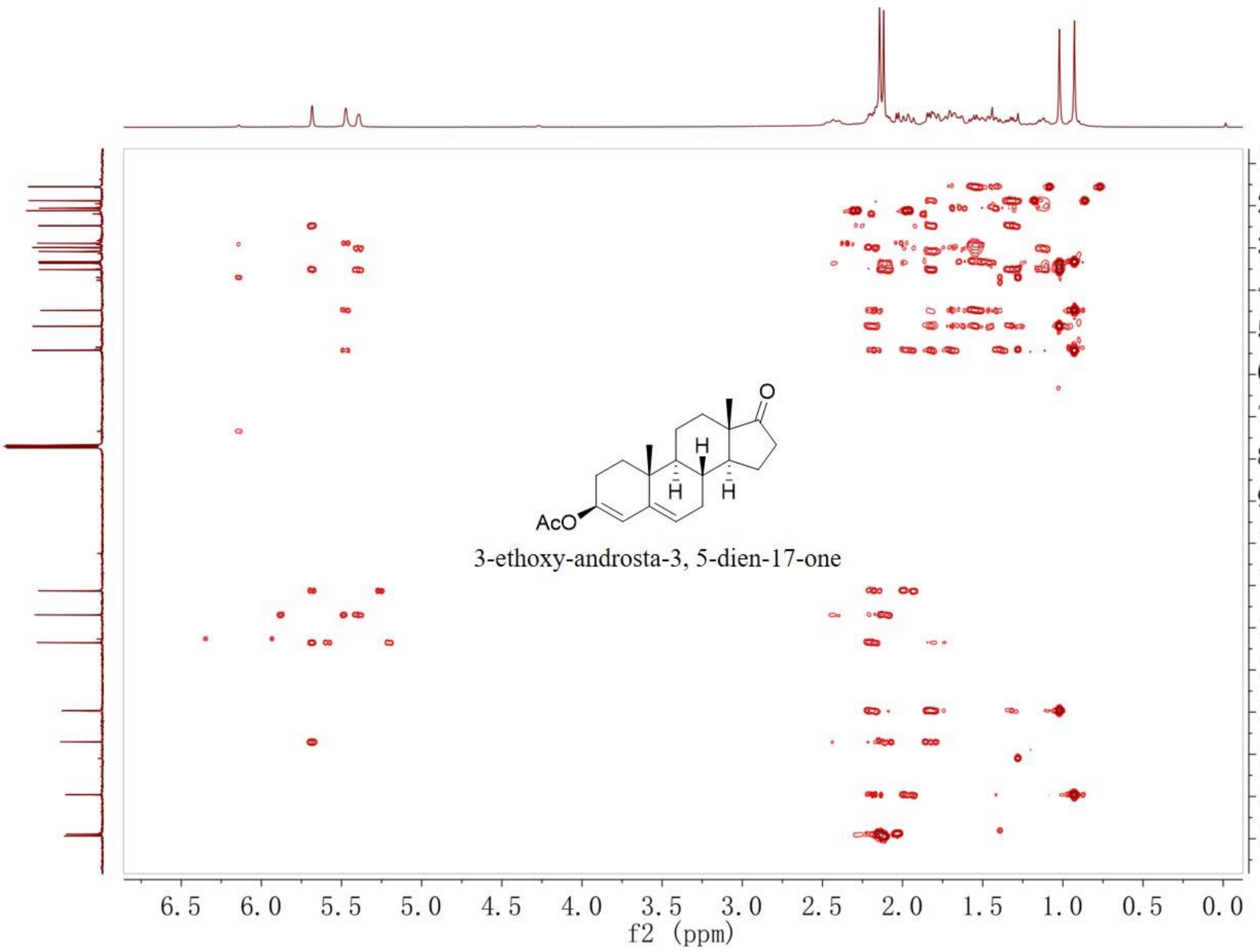

Figure. S6 HMBC spectrum of 3-ethoxy-androsta-3,5-dien-17-one in DMSO-d6 (400 MHz).

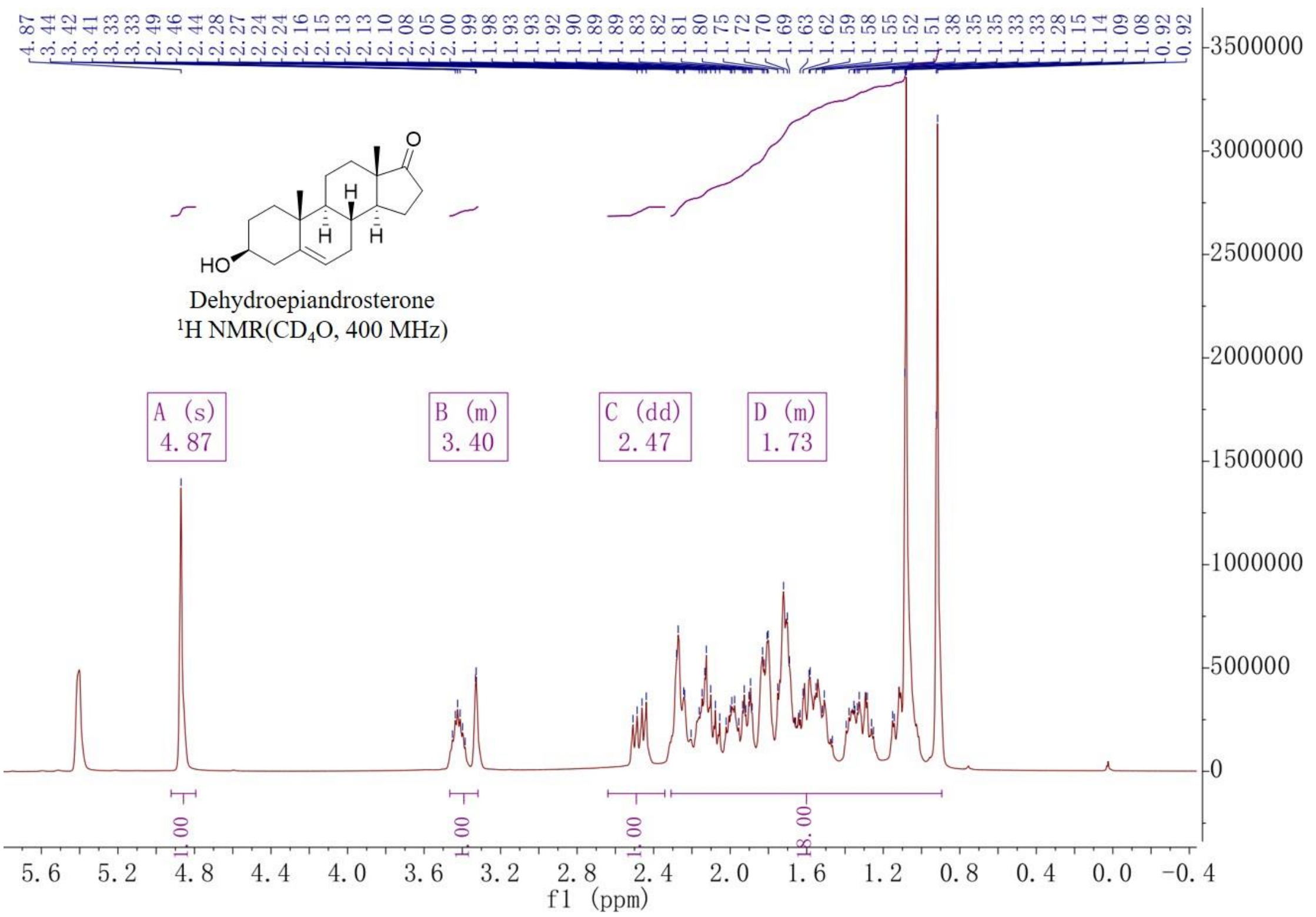

Figure. S7 ^{1}HNMR spectrum of dehydroepiandrosterone in Methanol-d4 (400 MHz).

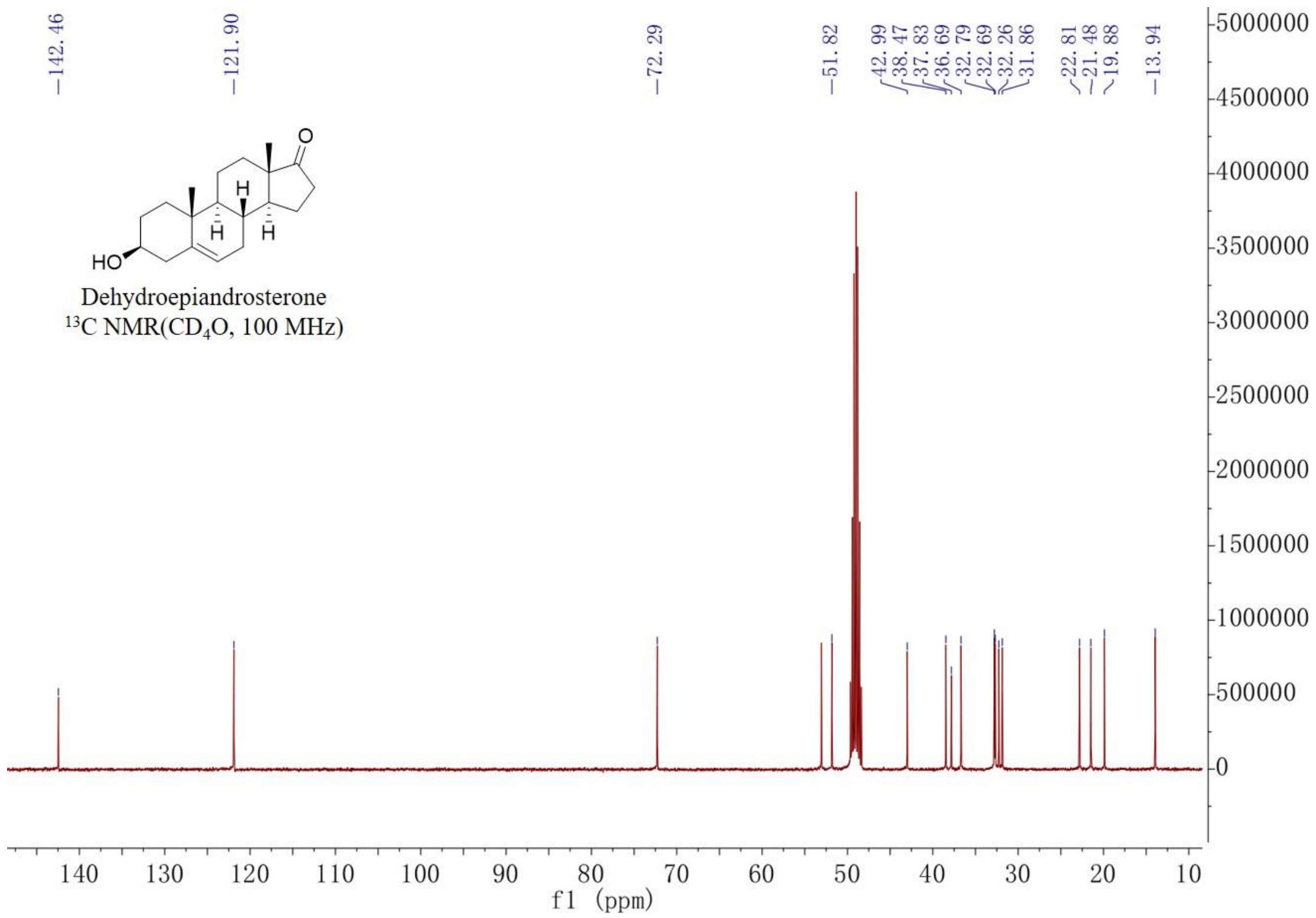

Figure. S8 ^{13}CNMR spectrum of dehydroepiandrosterone in Methanol-d4 (100 MHz).

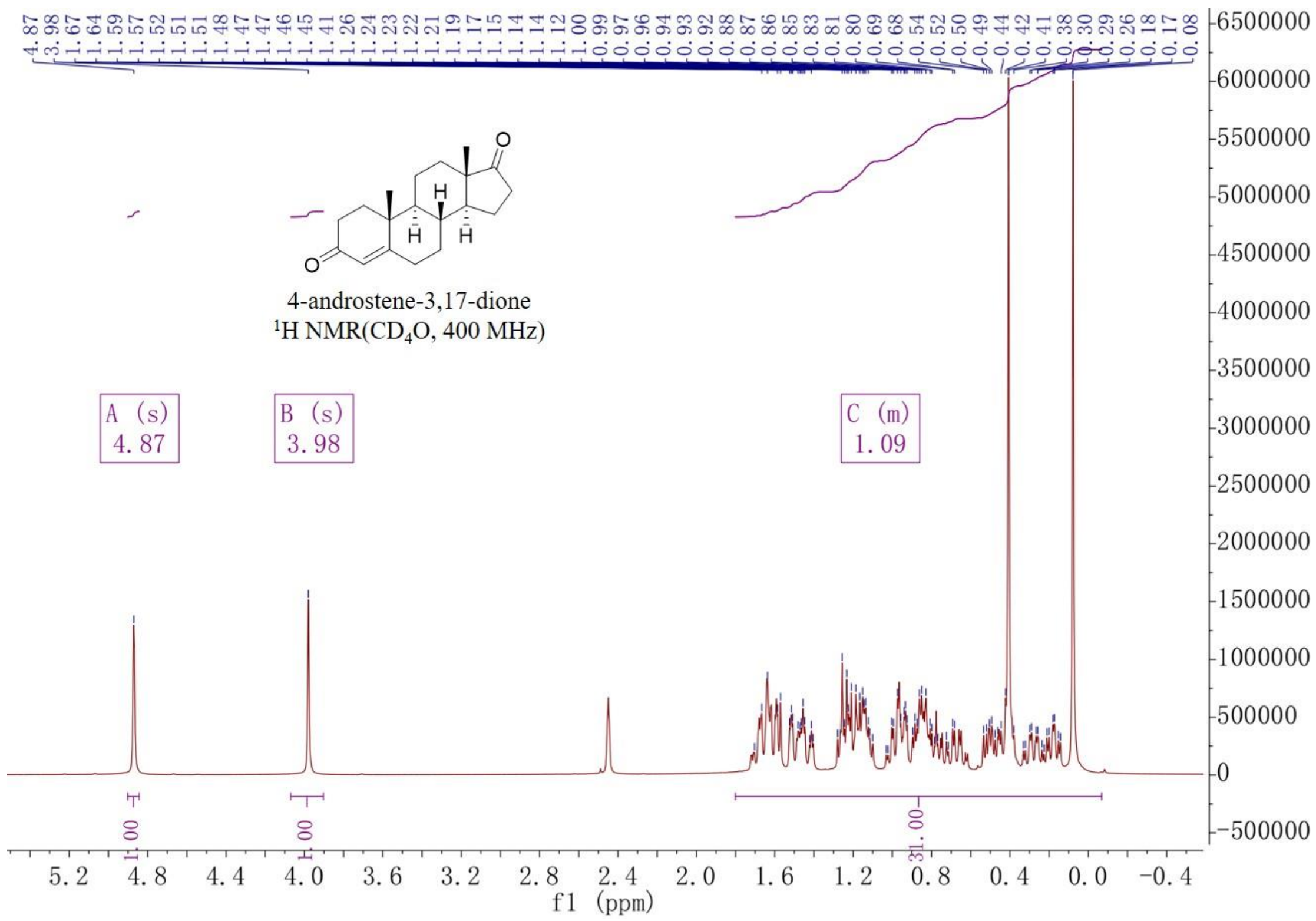

Figure. S9 ^{1}HNMR spectrum of 4-androstene-3,17-dione in Methanol-d4 (400 MHz).

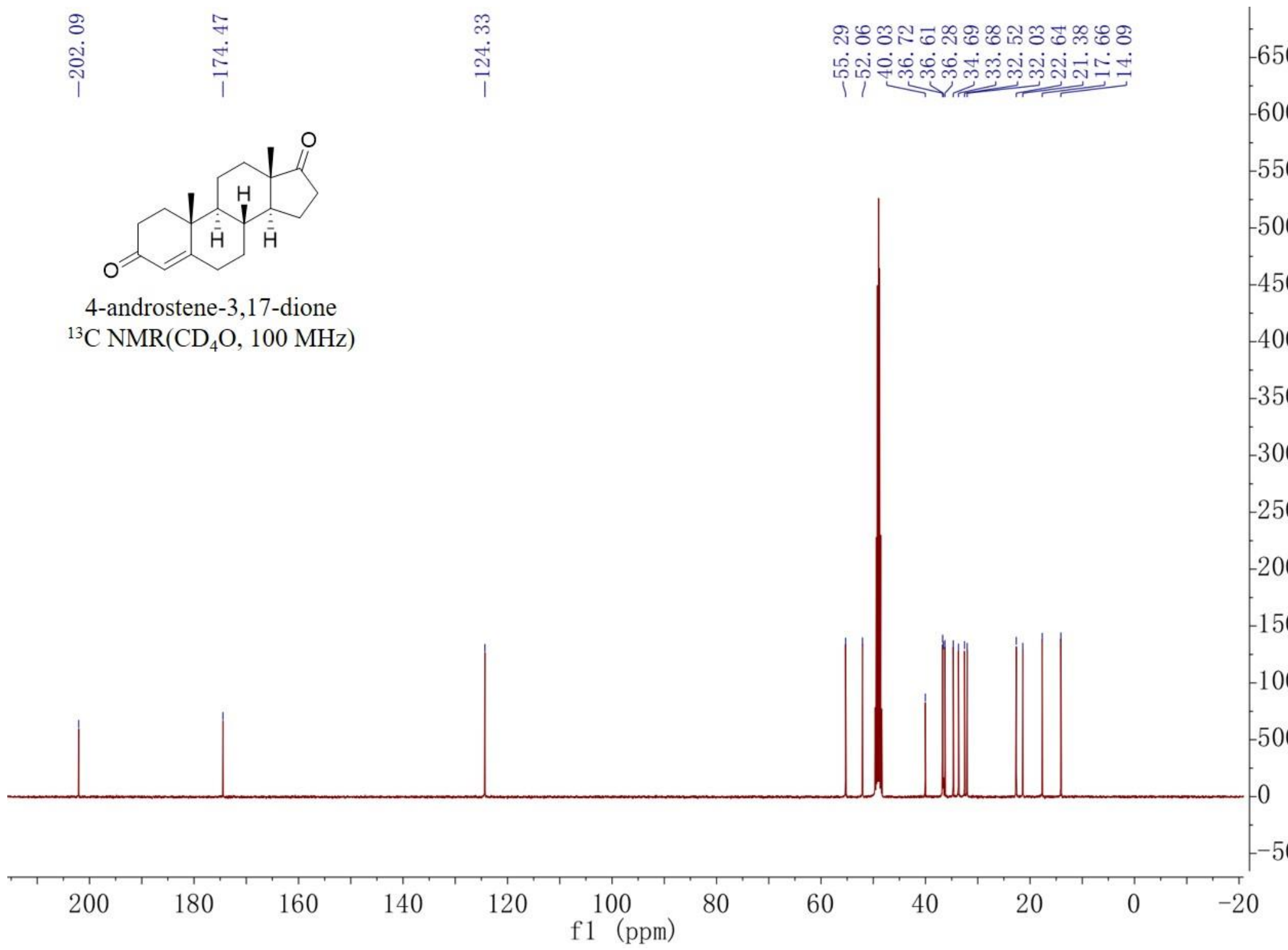

Figure. S10 ^{13}CNMR spectrum of 4-androstene-3,17-dione in Methanol-d4 (100 MHz).
